# Profiling the human intestinal environment under physiological conditions

**DOI:** 10.1038/s41586-023-05989-7

**Published:** 2023-05-10

**Authors:** Dari Shalon, Rebecca Neal Culver, Jessica A. Grembi, Jacob Folz, Peter V. Treit, Handuo Shi, Florian A. Rosenberger, Les Dethlefsen, Xiandong Meng, Eitan Yaffe, Andrés Aranda-Díaz, Philipp E. Geyer, Johannes B. Mueller-Reif, Sean Spencer, Andrew D. Patterson, George Triadafilopoulos, Susan P. Holmes, Matthias Mann, Oliver Fiehn, David A. Relman, Kerwyn Casey Huang

**Affiliations:** 1 Envivo Bio, Inc., San Francisco, CA USA; 2 Department of Genetics, Stanford University School of Medicine, Stanford, CA USA; 3 Department of Medicine, Stanford University School of Medicine, Stanford, CA USA; 4 West Coast Metabolomics Center, University of California, Davis, Davis, CA USA; 5 Department of Proteomics and Signal Transduction, Max-Planck Institute of Biochemistry, Martinsried, Germany; 6 Department of Microbiology and Immunology, Stanford University School of Medicine, Stanford, CA USA; 7 Department of Bioengineering, Stanford University, Stanford, CA USA; 8 Chan Zuckerberg Biohub, San Francisco, CA USA; 9 Division of Gastroenterology and Hepatology, Stanford University School of Medicine, Stanford, CA USA; 10 Department of Veterinary and Biomedical Sciences, Pennsylvania State University, University Park, PA USA; 11 Silicon Valley Neurogastroenterology and Motility Center, Mountain View, CA USA; 12 Department of Statistics, Stanford University, Stanford, CA USA; 13 Department of Food Science and Technology, University of California, Davis, Davis, CA USA; 14 Infectious Diseases Section, Veterans Affairs Palo Alto Health Care System, Palo Alto, CA USA

**Keywords:** Microbiome, Metabolomics

## Abstract

The spatiotemporal structure of the human microbiome^1,2^, proteome^3^ and metabolome^4,5^ reflects and determines regional intestinal physiology and may have implications for disease^6^. Yet, little is known about the distribution of microorganisms, their environment and their biochemical activity in the gut because of reliance on stool samples and limited access to only some regions of the gut using endoscopy in fasting or sedated individuals^7^. To address these deficiencies, we developed an ingestible device that collects samples from multiple regions of the human intestinal tract during normal digestion. Collection of 240 intestinal samples from 15 healthy individuals using the device and subsequent multi-omics analyses identified significant differences between bacteria, phages, host proteins and metabolites in the intestines versus stool. Certain microbial taxa were differentially enriched and prophage induction was more prevalent in the intestines than in stool. The host proteome and bile acid profiles varied along the intestines and were highly distinct from those of stool. Correlations between gradients in bile acid concentrations and microbial abundance predicted species that altered the bile acid pool through deconjugation. Furthermore, microbially conjugated bile acid concentrations exhibited amino acid-dependent trends that were not apparent in stool. Overall, non-invasive, longitudinal profiling of microorganisms, proteins and bile acids along the intestinal tract under physiological conditions can help elucidate the roles of the gut microbiome and metabolome in human physiology and disease.

## Abstract (web summary)

Variations in microbial composition, phage induction, antimicrobial resistance genes and bile acid profiles are identified by using an ingestible device for site-specific sampling along the intestines.

## Main

The human intestinal tract harbours the vast majority of microorganisms residing in or on our bodies^1^; their genetic content and biochemical transformation capabilities are hundreds of times larger than those encoded by the human genome^8^. Humans depend on their gut microorganisms for food digestion, immune system regulation and protection against pathogens, among other critical functions^1^. An important yet often overlooked aspect of the gut is regional heterogeneity and how it impacts local physiology^9^. Because of difficulties in accessing and sampling the intestinal tract, stool has been the main source of information for human gut microbiome studies^10^. However, stool reflects waste products and downstream effluent, within which regional variation is lost. For example, key metabolites such as bile acids are altered upstream by microbial transformations and then substantially absorbed by the host before excretion^4^. The regions of the gut distal to the stomach (duodenum, jejunum, ileum and colon) differ markedly in nutrient availability, pH, oxygen partial pressure, mucosal structure and flow rate^7^. As a result, distinct microbial communities with specialized functions, metabolomes, immune niches and proteomes are present in each intestinal region^3,4,11^. Thus, deeper understanding of how gut microorganisms impact human physiology and vice versa requires local sampling of the gut microbiome and its chemical environment in natural, unperturbed states.

Historically, sampling the human intestinal tract without disturbance or contamination has been challenging^10^. We recently discovered substantial regional variability in microbiota composition across spatial scales of only a few inches throughout the intestines of deceased organ donors^2^. However, organ donors have typically been treated with antibiotics, and, even in cases in which the intestinal tract has been sampled immediately after cessation of life support, the gut is often ischaemic or necrotic. Duodenal sampling from live individuals using upper endoscopy has a high probability of inadvertent contamination from oral, oesophageal or gastric contents. Endoscopic access to the mid-jejunum requires a ~2-h procedure involving general anaesthesia or sedation, performed under fasting^12,13^. Alternatively, a stoma from exteriorization of the ileum through the abdominal wall can provide intestinal samples, but this procedure is invasive and reflects altered gut anatomy and physiology, at a single location^14^. Despite the important effects on the microbiome and signalling properties of bile acids, studies on their chemical diversity and concentrations have relied on non-representative measurements of the few percent of bile acids in stool or the fraction of a percent in blood. Previously developed ingestible devices for sampling the human intestinal tract have important limitations such as complex electronics^15^, large size that risks device retention^15^ or insufficient sampling volume for multi-omics analyses^16^. pH profiles, peristalsis, diet, physiology, gastrointestinal disorders and key metabolites such as bile acids^17^ differ markedly between humans and animals^18^, making human studies most relevant to human physiology and disease.

To measure microbial, viral, proteomic and bile acid profiles within the human intestines during normal digestion, we developed and evaluated a capsule device that collects luminal contents from the small intestine or ascending colon. The expanding bladder and lack of internal structure in our device allowed ~400 µl of liquid to be retrieved, enabling multi-omic analyses. We report differences in microbiome composition, gene class abundance, prophage induction and the host proteome between the intestines and stool. We discovered gradients of microbially transformed bile acids along the intestinal tract and identified correlations between the abundance of microbially modified bile acids and specific gut bacterial species. In a separate manuscript, we combined five metabolomics assays to identify spatial and temporal differences between stool and intestinal metabolomes, including diet-derived compounds and microbially linked metabolites such as sulfonolipids and fatty acid esters of hydroxy fatty acid lipids^19^. These discoveries illuminate biological properties of the intestinal tract that are inaccessible from stool or endoscopic sampling.

## Device for sampling the human intestines

**Fig. 1:**
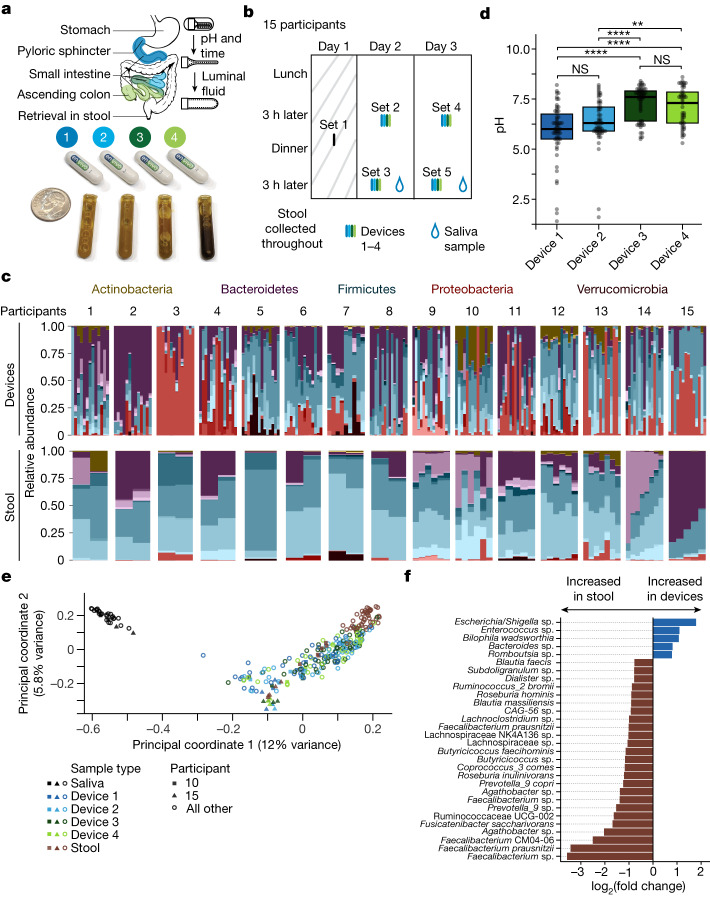
Devices enable longitudinal sampling of the human intestine. **a**, Overview of the intended sampling locations (top) of the four device types in packaged form for ingestion (middle) and as full collection bladders containing intestinal samples after retrieval from stool (bottom). A US dime is included for scale. Top right, the device contains a folded bladder capped with a one-way valve within a capsule with an enteric coating, which dissolves once the designated pH has been reached, enabling the bladder to unfold and draw in up to 400 µl of luminal fluid. **b**, Timeline for the collection of saliva, intestinal and stool samples from 15 healthy adults. Set 1 devices were not used for analyses. **c**, Family-level relative abundance for each sample by participant and location (*n* = 268). The colour of the ASV indicates the phylum, and the gradient of a given colour represents different families within the phylum. Only 16S rRNA gene ASVs with ≥3 reads in ≥5% of device and stool samples were used (*n* = 399 ASVs). **d**, The pH of the contents in devices designed to open at locations spanning the proximal to distal intestinal tract exhibited the expected increasing trend. Points represent individual devices (*n* = 218). *P* values from top to bottom: 0.018, 1.1 × 10^–4^, 5.5 × 10^−5^, 8.6 × 10^−8^, 1 and 0.19. Boxplots show the median value and the first and third quartiles. NS, not significant; **P ≤ 0.01, ****P ≤ 0.0001, Bonferroni-adjusted two-sided Wilcoxon rank-sum test. **e**, PCoA based on Canberra distance between microbial communities (*n* = 297). Read counts were log_2_ transformed. Each point represents an individual sample and is coloured by the sample type (stool, saliva and device types 1–4). Filled squares and triangles identify two outlier participants (10 and 15) who had taken oral antibiotics in the 5 months before intestinal sampling. Only 16S rRNA gene ASVs with ≥3 reads in ≥5% of samples (including saliva) were used (*n* = 455 ASVs). **f**, ASVs with log_2_(fold change) > 0.75 between devices and stool that were significantly differentially abundant (*n* = 28 ASVs across *n* = 268 analysed samples; limma-voom was used to calculate differential expression after size factors were estimated and normalized using DESeq2; *P* < 0.05, Benjamini–Hochberg correction).

The sampling capsule is a single-use, passive device that collects fluid from the human intestines for ex vivo analysis. The device contains a collapsed collection bladder capped by a one-way valve inside a dissolvable capsule with an enteric coating (Fig. 1a). The enteric coating prevents contact between the collection bladder and the surrounding environment before entry into the intestines. The pH of the intestines typically rises from 4–6 in the duodenum to 7–8 in the ileum^15^. Once the device reaches a pre-set pH sufficient to dissolve the enteric coating, the collection bladder expands and draws in luminal contents through the one-way valve. To sample from four distinct regions of the intestinal tract, four devices were ingested as a set after an individual ate a meal of their choosing, wherein different device types in a set were designed to open at different, progressively higher pH levels. Device type 4 included a time-delay coating to bias collection towards the ascending colon where the pH typically drops relative to the terminal ileum^15^ (Methods and Fig. 1a). Each device collects up to 400 µl of luminal contents; bacterial density is higher in the lumen than at or within the mucosa^20^, most mucosa-associated bacteria are represented in the luminal contents^21^ and many metabolites of interest are in the lumen. After the bladder fills, the one-way valve prevents further entrance of liquid. The ingested devices are recovered from stool, and collected samples are extracted for analysis. These devices provide unique potential for multi-region collection of microorganisms and metabolites within the intestines during normal digestion.

**Extended Data Fig. 1:**
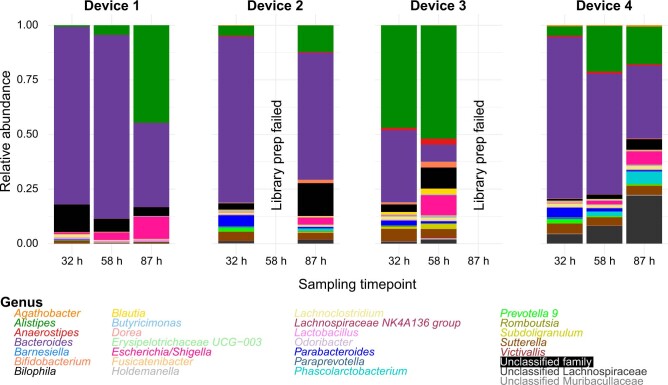
Changes in intestinal microbiota composition during sample incubation. Devices were collected from a bowel movement of a single subject 32 h after ingestion and placed immediately into an anaerobic chamber at 37 °C. Samples were collected from each device immediately (32 h) and again at 58 h and 87 h. Samples were then prepared for 16S rRNA gene sequencing. Genus-level relative abundance is shown for all ASVs with read count ≥15 in any single sample.

We first sought to confirm whether the devices could be targeted to specific intestinal locations and would progress through the intestinal tract without contamination. In a feasibility study, we connected devices targeting the jejunum and ascending colon to a capsule endoscope and visualized successful in vivo sampling in a human (Supplementary Video 1). To assess potential effects of incubation of the collected microorganisms in the device while it transited through the gut, we retrieved and incubated a set of four devices from a single bowel movement in an anaerobic chamber at 37 °C for up to 87 h (Methods). We found that major changes in microbiota composition did not occur in devices with a transit (incubation) time of ~58 h or less (Extended Data Fig. 1). Within these experimental limitations, we demonstrate below that microorganisms and metabolites display longitudinal gradients along the intestine and are highly distinct from the contents of stool samples.

## Spatially distinct microbial communities

To assess compositional and functional differences within the intestinal microbiome, we carried out a clinical study with 15 healthy human participants (Supplementary Table 1). First, a single device was swallowed and retrieved to ensure that no complications arose during device passage through the gut (set 1; Fig. 1b); the contents of these devices were not analysed. Subsequently, sets of four devices (with each device type within a set having a different enteric coating) were ingested twice daily (3 h after lunch and 3 h after dinner) on two consecutive days (sets 2–5; Fig. 1b). All participants consumed their normal diets and kept a food log. All devices safely exited all participants and were successfully retrieved. No adverse events were reported. Participants collected contemporaneous saliva samples (*n* = 2, one on each day before ingesting devices 3 h after dinner) and 2–8 stool samples on or around the days when devices were recovered (Fig. 1b).

**Extended Data Fig. 2:**
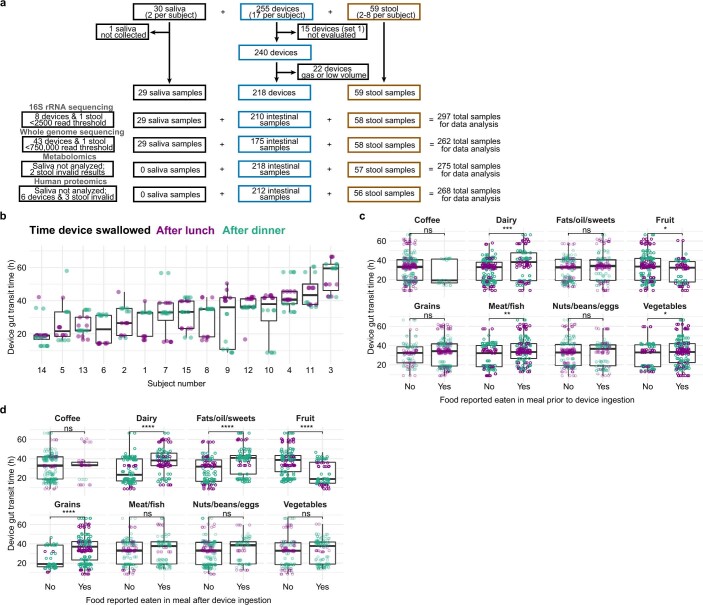
Total gut transit time of devices varied across subjects and diets. a) Subjects (*n* = 15) enrolled in the study ingested a total of 17 intestinal sampling devices each (set 1 consisted of a single device used as a safety test to ensure safe passage through the intestines). Subjects were also asked to provide two saliva samples and collect stool until all intestinal sampling devices were retrieved. Between two and eight stool samples from each subject were used for analysis. b) Device (*n* = 240) gut transit time was variable across subjects. Some subjects displayed differences in transit time dependent on the time of day the device was ingested. c) Device gut transit time varied according to certain types of food consumed in the meal prior to device ingestion (i.e., the food with which the devices presumably transited into the small intestines). *P*-values from left to right, top to bottom: 0.18, 0.00091, 0.24, 0.17, 0.29, 0.0049, 0.57, and 0.27. d) Device gut transit time varied according to the type of food consumed in the meal after devices were swallowed (i.e., the food that likely influenced gut motility while devices were passing through the large intestines). *P*-values from left to right, top to bottom: 0.68, 1.4 × 10^−7^, 1.1 × 10^−7^, 7.6 × 10^−11^, 7 × 10^−7^, 0.49, 0.25, and 0.3. Boxplots show the median and 1^st^ and 3^rd^ quartiles. Each dot is a device sample (b–d; *n* = 60 each for device types 1–4). ns: not significant, *: *P* ≤ 0.05, **: *P* ≤ 0.01, ***: *P* ≤ 0.001, ****: *P* ≤ 0.0001, Bonferroni-corrected two-sided Wilcoxon rank-sum test. Alcohol consumption and diet contents were not restricted. Subjects swallowed devices 3 h after lunch and dinner and were instructed not to consume any additional foods for at least 2 h after swallowing devices.

We obtained sufficient sampling volume and 16S rRNA gene sequencing depth from 210 devices, 29 saliva samples and 58 stool samples (Extended Data Fig. 2a and Methods). The pH profiles of the samples collected by the four device types (Fig. 1d) reasonably matched previously published measurements of pH along the human intestines, with a general increase in pH from the proximal to distal small intestine followed by a decrease in the ascending colon^15^. The time between device ingestion and recovery ranged from 8 to 67 h (Extended Data Fig. 2b), in line with previous reports of broadly distributed transit times^15^. Given typical gastric emptying times and the 3-h post-meal interval before devices were swallowed, the devices probably entered the small intestine with the final contents of the preceding meal^22,23^. Nonetheless, the contents of the subsequent meal were more strongly associated with device transit time (Extended Data Fig. 2c,d).

**Extended Data Fig. 3:**
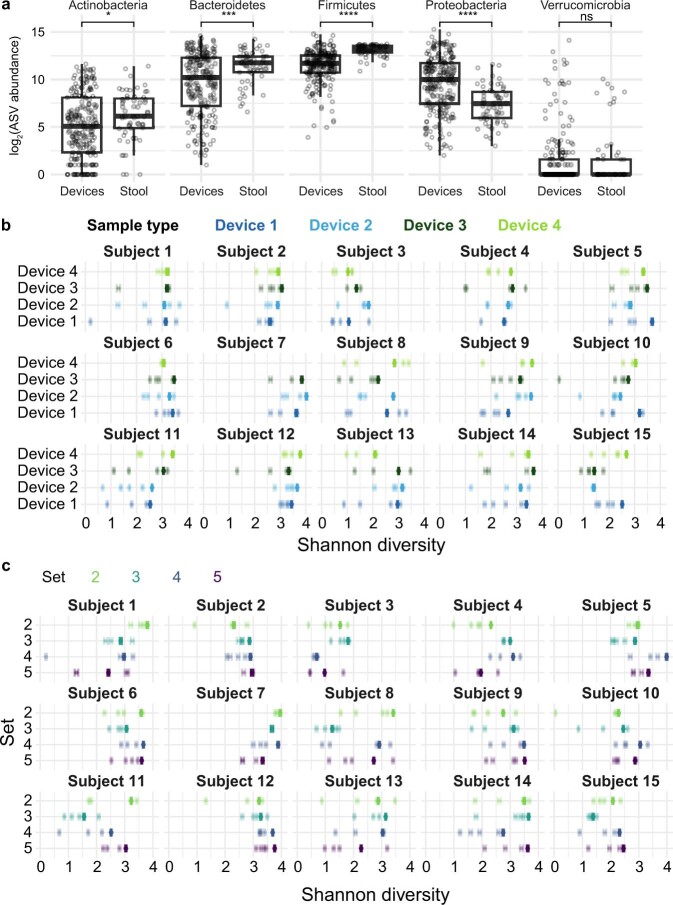
Diversity in phylum abundances and alpha and gamma diversity implicate a temporally and spatially heterogeneous intestinal tract. a) Summed log_2_(read count) at the phylum level are shown for each sample by location. *P*-values from left to right: 0.57, 0.0035, 2.4 × 10^−15^, 5.7 × 10^−7^, and 0.83. ASVs that did not have read count ≥3 in 5% of samples were ignored. Boxplots show the median and 1^st^ and 3^rd^ quartiles. Each dot represents a sample (*n* = 210 and 58 for devices and stool, respectively). ns: not significant, *: *P* ≤ 0.05, **: *P* ≤ 0.01, ***: *P* ≤ 0.001, ****: *P* ≤ 0.0001, Bonferroni-corrected two-sided Wilcoxon rank-sum test. b, c) The Shannon diversity of individual devices (*n* = 210 devices; alpha diversity, lighter points) was generally lower than that collectively of all devices from a single set (gamma diversity, bold points), indicating high spatial variation. Each subject (#1–#15) is shown separately. To ensure equal read depths for accurate comparisons, all intestinal samples were rarefied to the minimum sequencing depth of any device from that subject. Mean values and 95% confidence intervals for alpha and gamma diversity estimates were obtained by repeating the rarefaction procedure 1000 times.

A principal coordinate analysis (PCoA) based on Canberra distance identified location along the intestinal tract and across disparate sample types (saliva, intestines and stool) as an important latent variable. Saliva samples were significantly segregated from intestinal and stool samples across all participants (PERMANOVA, *P* = 0.001; Fig. 1e), indicating that the composition of the contents of all devices was distinct from the composition of the oral microbiota. Furthermore, we identified two participants (10 and 15; Fig. 1e) whose stool, and to some degree intestinal samples, clustered separately. On follow-up questioning, these participants reported taking antibiotics within the past 1 month (participant 10) and 5 months (participant 15). When considering each of the 15 participants individually, 23% ± 10% (137 ± 70 of 582 ± 85) of the amplicon sequence variants (ASVs, a proxy for species) detected in the devices were not detected in the participant’s saliva or stool; the median relative abundance of these 137 ASVs was low (<0.4%). Similarly, 12% ± 8% of the ASVs in stool were not detected in the participant’s intestinal samples, and the median relative abundance of these ASVs was low (<0.6%) in all but one outlier participant (participant 3) whose intestinal samples were dominated by a single species (and hence many abundant ASVs in stool were not detected in the intestinal samples). In line with previous studies^24^, we observed higher relative abundance of the Proteobacteria phylum in the intestines than in stool (Extended Data Fig. 3a), including a *Bilophila wadsworthia* ASV, consistent with previous reports of *B.* *wadsworthia*’s key role in the small intestine^25^. Four additional ASVs, from the *Escherichia*/*Shigella*, *Enterococcus*, *Bacteroides* and *Romboutsia* genera, were significantly more abundant (adjusted *P* < 0.05 and log_2_(fold change) > 0.75) in intestinal samples than in stool (Fig. 1f). The *Romboutsia* genus was recently named following isolation of a species from rat ileal digesta^26^, in line with this genus having a niche in the small intestine.

**Fig. 2:**
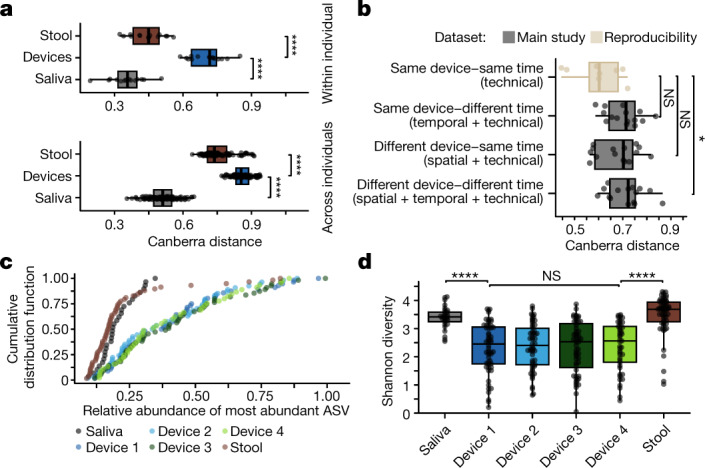
Microbiota variation across device types suggests patchy structure. **a**, Microbiota composition varied significantly more between intestinal samples than between stool samples (*P* = 1.5 × 10^−7^ within participants and *P* = 2.3 × 10^−22^ across participants) or between saliva samples (*P* = 1.5 × 10^−7^ within participants and *P* = 3.5 × 10^−35^ across participants). Top, each point is the mean pairwise Canberra distance between all samples for a participant (*n* = 14, 15 and 14 for stool, devices and saliva, respectively). Bottom, each point is the mean of all pairwise comparisons between all samples from any two participants (*n* = 105, 105 and 105 for stool, devices and saliva, respectively). **b**, Combinations of spatial, temporal and technical (*n* = 15 each) variability in the microbiota composition of intestinal samples (gray) were higher than in technical replicates (*n* = 8; light brown) in which one participant swallowed four of the same device type simultaneously (the participant did so twice for each of the four device types). Each point represents the mean pairwise Canberra distance between intestinal samples from the same participant. Microbial communities from devices of the same type ingested at the same time were more similar than those from devices of the same type ingested at different times, although this difference was not statistically robust (*P* = 0.058) given the small number of observations. **c**, Devices were more likely to be dominated by a single ASV as compared with stool or saliva. Each point is a single sample (*n* = 29 for saliva, *n* = 56, 54, 55 and 45 for device types 1–4, respectively, and *n* = 58 for stool). **d**, The Shannon diversity of saliva and stool samples was higher than that of intestinal samples (saliva to device type 1, *P* = 5.3 × 10^−7^; device type 4 to stool, *P* = 2.9 × 10^−9^). Each point is a single sample (*n* = 29 for saliva, *n* = 56, 54, 55 and 45 for device types 1–4, respectively, and *n* = 58 for stool). Boxplots show the median value and the first and third quartiles. *P ≤ 0.05, ****P ≤ 0.0001, Bonferroni-adjusted two-sided Wilcoxon rank-sum test. Canberra distances for **a**,**b** were computed from log_2_-transformed read counts of 16S rRNA gene ASVs with read count ≥3 in ≥5% of samples (including all repeatability samples) (*n* = 446).

We observed more intra-individual microbiota variability among intestinal samples than among stool or saliva samples (Fig. 2a), suggesting that the devices collect from a more heterogenous habitat. Although device types 1–4 were designed to sample the intestines longitudinally, comparisons of microbiota composition among devices of the same type but swallowed at different times are potentially confounded by variability in meal contents, periprandial neurohormonal variations, intestinal motility, pH and/or the intestinal microbiota itself. We therefore assessed technical and biological variability by having one participant ingest four devices of the same type simultaneously; this procedure was repeated twice for each device of types 1–4 over the course of 2 months. Devices of the same type ingested at the same time contained more similar microbial communities than devices of the same type ingested at different times (Fig. 2b). The increased variance in microbiota composition due to this temporal variability is comparable to the variance due to spatial variability along the intestine, as assessed using sets of four devices of distinct types ingested at the same time (Fig. 2b). Moreover, intestinal samples (unlike saliva or stool samples) were often dominated by a single ASV with relative abundance of >40% (Fig. 2c). Consequently, individual intestinal samples contained communities with lower alpha diversity relative to the intra-individual diversity represented by all samples from a device of a certain type or by all samples from devices swallowed at the same time (Fig. 2d and Extended Data Fig. 3b,c). Thus, much of the higher variability across intestinal samples relative to stool is probably due to the dynamic and heterogeneous nature of the microbiota along the intestinal tract.

## Bacteria remain viable within devices

**Extended Data Fig. 4:**
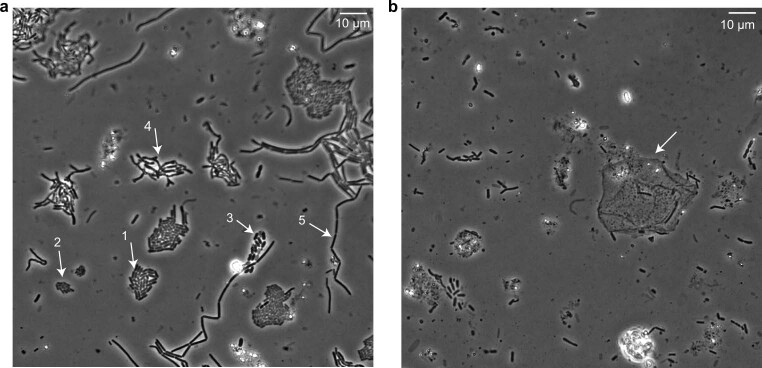
Growing cells recovered from devices collectively displayed a wide range of morphologies. a) A 2-µL sample was acquired from a single device and spotted onto an agarose pad with BHI medium. After 4 h of time-lapse imaging, growing cells displayed a wide range of morphological features, as highlighted by white arrows: 1. regular rods; 2. small rods; 3. wide rods; 4. branching; and 5. long/filamentous rods. Along with the sample shown, 3 other samples from devices ingested at the same time were imaged, and similar results were observed. b) Occasional human cells (white arrow) were observed during imaging.

To determine whether the intestinal microorganisms collected by the capsule devices were viable, participant 1 ingested a device designed to collect from the proximal region of the intestines. An aliquot of the sample was retrieved under anaerobic conditions ex vivo and placed on an agarose pad with nutrients. The pad was sealed to prevent oxygen from diffusing to the cells and subjected to time-lapse imaging (Methods). Over 4 h, 20–50% of cells resumed growth (Supplementary Video 2); a similar regrowth fraction was observed in anaerobic resuspensions of fresh stool (Supplementary Video 3), indicating that the devices preserve live bacteria to the same degree as seen with fresh stool. The growing cells recovered from the device collectively displayed a wide range of morphologies (Extended Data Fig. 4a), suggesting that regrowth is not heavily biased towards a few taxa. Supporting this conclusion, we used plating and flow cytometry to obtain a library of 456 isolates from several intestinal samples from participant 1 and 31 isolates from stool samples from participant 1, comprising at least 51 unique species across four phyla (Methods and Supplementary Table 2). In our time-lapse imaging, we also noted occasional human cells (~0.1% of cell count; Extended Data Fig. 4b) that were probably epithelial cells on the basis of their morphology, in line with the small fraction of metagenomic reads from these samples that mapped to the human genome. Taking these findings together, the devices enable culturomics experiments and may provide the opportunity to study host cells present in the lumen.

## Genetic variation along the intestines

**Extended Data Fig. 5:**
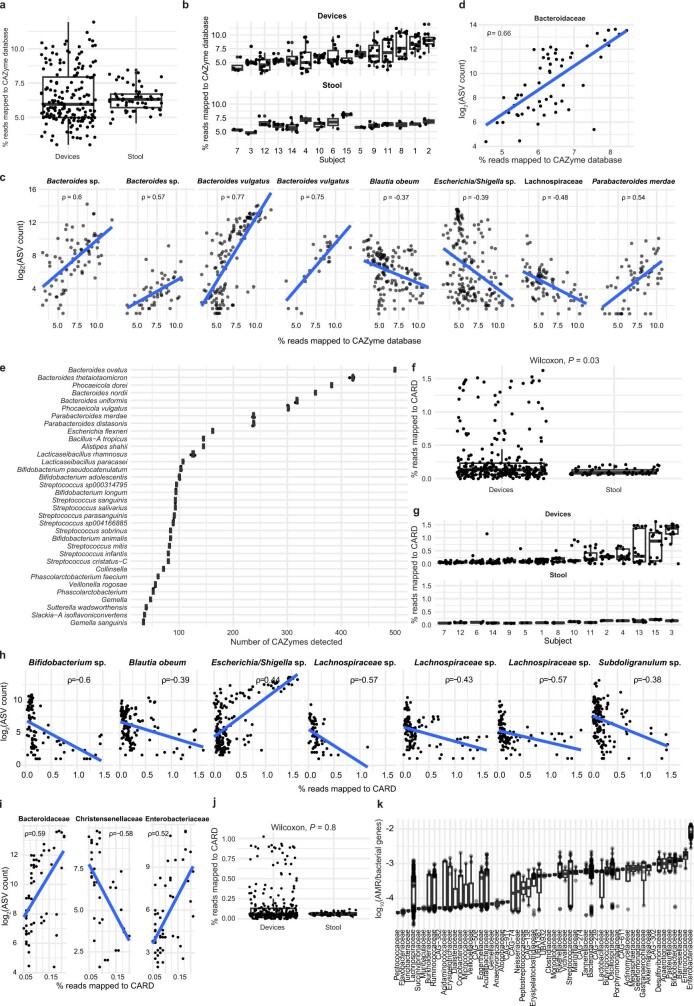
Percent of reads mapping to CAZymes and antimicrobial resistance genes vary across the intestinal tract and are driven by distinct taxa. a) The percent of reads that mapped to a database of CAZymes (Methods) was determined using metagenomic sequencing of each sample. b) Data from (a) separated by subject. c) In intestinal samples, the log_2_(read count) of eight ASVs was positively correlated (Spearman) with the percent of reads that mapped to the CAZyme database. Only ASVs with *P* < 0.001 are shown. *P*-values from left to right, top to bottom: 1.8 × 10^−6^, 7.2 × 10^−4^, 1.1 × 10^−24^, 3.7 × 10^−4^, 3.7 × 10^−4^, 2.6 × 10^−5^, 3.7 × 10^−4^, and 3.7 × 10^−4^. d) The log_2_(ASV read count summed over family members) of the Bacteroidaceae family was significantly correlated (Spearman; *P* = 6.4 × 10^−7^) with the percent of reads from stool samples that mapped to the CAZyme database. No other families had *P* < 0.01. e) The number of CAZymes identified in strains isolated from the intestinal tract of subject 1, organized by species. Each circle represents a single strain, and horizontal lines (mostly hidden by the circles) represent the median. f) The percent of reads that mapped to a database of AMR genes (CARD, Methods) was determined using metagenomics sequencing of each sample and was higher in devices compared with stool (*P* = 0.03). g) Data from (f) separated by subject. h) In intestinal samples, the log_2_(read count) of two ASVs was positively correlated (Spearman) with the percent of reads that mapped to CARD. Only ASVs with *P* < 0.001 are shown. *P*-values from left to right, top to bottom: 5.7 × 10^−8^, 1.5 × 10^−4^, 4.1 × 10^−7^, 2.5 × 10^−4^, 6.4 × 10^−5^, 2.0 × 10^−8^, and 2.1 × 10^−4^. i) In stool samples, the log_2_(ASV read count summed over family members) of families was significantly correlated (Spearman; *P* = 5.7×10^−5^, 1.5 × 10^−3^, and 7.1 × 10^−4^) with the percent of reads that mapped to a database of AMR genes. No other families had *P* < 0.001. j) The percent of reads that mapped to CARD ignoring all efflux pumps was similar between devices and stool (*P* = 0.8). k) log_10_(ratio of the number of AMR genes to bacterial genes) detected in each MAG, aggregated by family-level taxonomic assignment. Only MAGs with completion >75% and contamination <10% were included. In (a–d and f–j), each dot is a sample (*n* = 175 for devices and *n* = 58 for stool). All *P*-values reported are after Benjamini-Hochberg correction. Boxplots show the median and 1^st^ and 3^rd^ quartiles.

To evaluate functional differences between the intestinal and stool microbiota, we performed metagenomic sequencing on all device and stool samples (Methods). We obtained 696 dereplicated metagenome-assembled genomes (MAGs; >75% complete and <25% contamination) from these data (Methods and Supplementary Table 3), which enabled taxonomic identification for read-mapping applications. On the basis of the established role of the gut microbiota in carbohydrate degradation and its links to health and disease^27^, we first focused on carbohydrate active enzyme (CAZyme) gene abundance in each region. The percentage of reads that mapped to CAZymes in devices exhibited greater variance than in stool (Extended Data Fig. 5a,b). Within devices, CAZyme gene abundance was positively correlated with the relative abundance of five ASVs: two unnamed *Bacteroides* species, two *Bacteroides vulgatus* strains and *Parabacteroides merdae* (*P* < 0.001, Benjamini–Hochberg corrected; Extended Data Fig. 5c). The *B.* *vulgatus* strains exhibited the highest slope and strongest correlation (Spearman’s *ρ* = 0.77 and 0.75). By contrast, in stool, despite a correlation between the abundance of CAZyme genes and the Bacteroidaceae family (Extended Data Fig. 5d), there were no ASVs whose abundance correlated with CAZyme gene abundance, probably because of the greater evenness of the taxa observed in stool compared with intestinal samples (Fig. 2c).

To evaluate whether certain species explain CAZyme gene abundance in intestinal samples, we investigated the genomic content of our intestinal strain library of 456 isolates derived from device samples. Whole-genome sequencing of 74 phylogenetically diverse strains (completeness of >95%; Supplementary Table 2) from this library showed that the 35 members of the Bacteroidetes phylum typically contained more CAZyme genes than members of other phyla (Extended Data Fig. 5e). The dataset included ten *Parabacteroides* strains (eight *Parabacteroides distasonis* and two *P.* *merdae*). Each CAZyme gene was annotated with a CAZyme enzyme class and family to give a putative functional category. The CAZymes detected in the *P.* *merdae* strains were assigned to a mean of 107.5 unique CAZyme functional categories out of a mean of 237.5 CAZymes, and *P.* *distasonis* enzymes were assigned to 95 unique CAZyme functional categories out of a mean of 237.5 CAZymes; thus, *P.* *distasonis* strains appear to contain greater redundancy than *P.* *merdae* strains (Supplementary Table 4). Furthermore, *P.* *merdae* strains contained seven additional unique CAZyme functional categories in the glycoside hydrolase family and five additional unique polysaccharide lyase functional categories compared with *P.* *distasonis* strains (Supplementary Table 4). We also investigated five strains of *B.* *vulgatus*: each possessed 301 or 302 CAZyme genes representing 131 unique functional categories, more than in any other non-*Bacteroides* isolate (Extended Data Fig. 5e and Supplementary Table 4). However, *B.* *vulgatus* was the *Bacteroides* species with the fewest CAZyme genes (Extended Data Fig. 5e and Supplementary Table 4), indicating that factors other than CAZyme abundance influence the dominance of *B.* *vulgatus* over other *Bacteroides* species in the intestines. These differences in CAZyme gene abundance and functional categories are an important consideration for how diet drives the growth of certain bacteria in the gastrointestinal tract and for which by-products of carbohydrate degradation may be available to the host.

Given the substantial differences in microbiota compositions in the two participants who reported recently taking antibiotics compared with the other participants (Fig. 1e), we sought to determine whether metagenomic sequencing data could identify differences in antimicrobial resistance (AMR) potential. We focused on 6,453 AMR gene ontologies identified by the RGI algorithm on the basis of the Comprehensive Antibiotic Resistance Database (CARD, which uses a rigorously curated collection of peer-reviewed resistance determinants^28^; Methods) and calculated the percentage of reads in each sample that aligned to CARD. There were 9,596 AMR genes detected across all samples; 3,590 of these were unique and ≥90% the length of a reference AMR gene. By mapping reads from all samples to this database of 3,590 AMR genes, we found that intestinal samples had significantly higher percentages of reads that mapped to the CARD database than stool samples (*P* = 0.03, Wilcoxon rank-sum test; Extended Data Fig. 5f). In general, the frequency of AMR genes in stool was similar across participants, although some participants exhibited ~2- to 3-fold-higher mean frequencies of putative AMR genes in their intestinal samples than other participants (Extended Data Fig. 5g). Further analyses (Methods) demonstrated that the abundance of *Escherichia*/*Shigella* species may result in larger reservoirs of AMR genes, particularly efflux-related genes, in the intestinal tract than was previously appreciated when assessing AMR in stool samples.

## Increased prophage induction in intestines

**Fig. 3:**
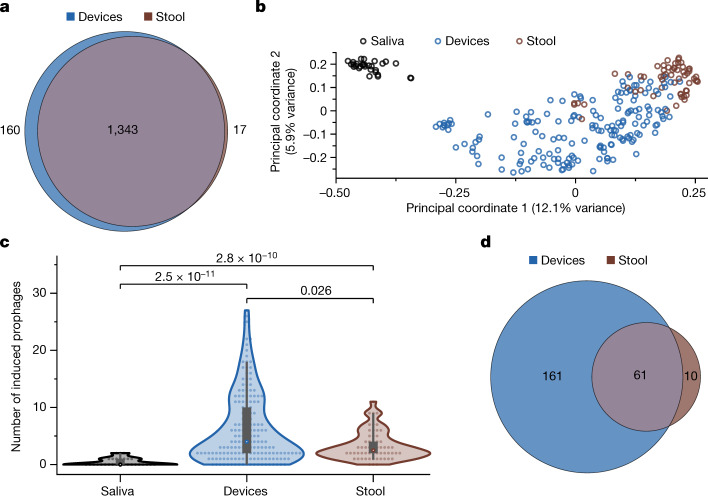
Prophage induction is more frequent in the intestines than in stool. **a**, Stool and intestinal samples share most vOTUs. Only vOTUs at a depth of ≥1.0 were included. **b**, PCoA based on Canberra distance between profiles of vOTUs detected in samples coloured on the basis of sample type. **c**, Intestinal samples contained significantly higher numbers of induced prophages than stool (*P* = 0.026) or saliva (*P* = 2.5 × 10^−11^) samples. *n* = 29, 172 and 58 for saliva, intestinal and stool samples, respectively. *P* values are from a two-sided Wilcoxon rank-sum test. Density boxplots show the median value and the first and third quartiles. **d**, Most prophages induced in stool samples are also induced in the intestines, but many other induced prophages are unique to intestinal samples.

**Extended Data Fig. 6:**
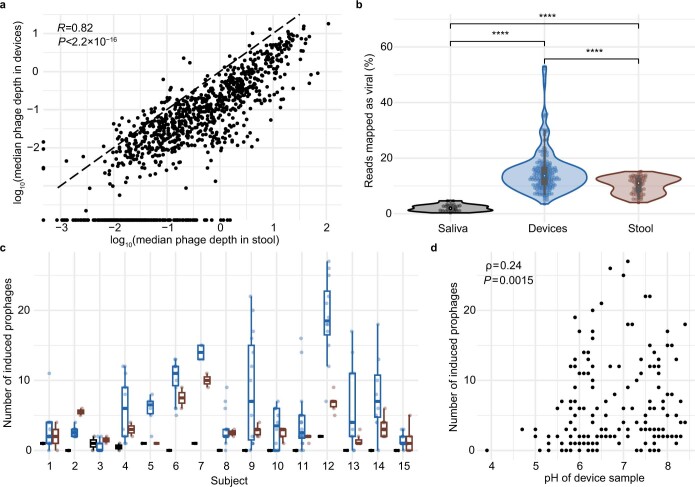
Intestinal samples have higher bacteriophage load. a) The median sequencing depth of vOTUs (*n* = 1343) in intestinal samples was highly correlated with depth in stool samples (Pearson; *P* < 2.2 × 10^−16^). 481 vOTUs appeared in less than half of the intestinal samples and hence the median was 0 (represented as 10^−4^). b) Intestinal samples had significantly higher read fraction mapping to vOTUs. *n* = 29, 172, and 58 for saliva, intestinal, and stool samples, respectively. *P*-values are from two-tailed Student’s t-tests. *P*-values from top to bottom, left to right: 4.7 × 10^−14^, 2.2 × 10^−16^, 2.3 × 10^−7^. c) The number of induced prophages was generally higher in intestinal samples across subjects and was lowest in saliva samples. d) pH was correlated with the number of induced prophages in intestinal samples (Spearman; *P* = 0.0015).

Our metagenomics dataset also provided an opportunity to investigate the viral component of the intestinal microbiota. From the assembled contigs, we identified 1,607 viral operational taxonomic units (vOTUs) with >50% completeness, of which 629 were integrated prophages (Methods). Of these vOTUs, 83% (1,343/1,607) were present in both stool and intestinal samples (Fig. 3a), indicating that the intestines and stool have similar viromes. The abundance of these vOTUs as determined by read mapping was generally correlated between intestinal and stool samples (Extended Data Fig. 6a), although the intestinal samples had higher viral read mapping fractions (Extended Data Fig. 6b), perhaps owing to lower bacterial densities^1^. Viromes were more similar between stool and intestinal samples from the same participant (Jaccard distance of 0.40 ± 0.14, mean ± s.d.) than between stool (0.58 ± 0.09) or intestinal (0.62 ± 0.10) samples from different participants (*P* < 10^−10^ in both cases, two-tailed Student’s *t*-test), and PCoA of the viromes (Fig. 3b) showed similar clustering as with the microbiota (Fig. 1e).

Quantification of prophage induction events based on the ratio of coverage of the viral and bacterial regions of the contig (Methods) showed significantly higher numbers of induced prophages in intestinal compared with stool samples (Fig. 3c). Most prophages (61/71) that were induced in the stool samples were also induced in the intestine; by contrast, 161 of the 222 induced prophages in intestinal samples were not observed in stool (Fig. 3d). Similar differences in prophage induction between intestinal and stool samples were observed in most participants (Extended Data Fig. 6c).

Of the contigs annotated as prophage, 279 of 629 were associated with a MAG and hence could be readily assigned taxonomy (Supplementary Table 5). Of the 328 induced prophages, the taxonomy of 138 could be assigned reliably and was collectively phylogenetically diverse, including Actinobacteria, Proteobacteria, Firmicutes and Bacteroidetes. The induced prophages were not strongly biased towards any MAG or taxon, with each MAG possessing a median of one induced prophage. Taxonomy was annotated for 141 (of 301) dormant phages, with each MAG possessing a median of one dormant phage, and these annotations were similarly diverse as the induced prophages. The number of prophage induction events was correlated with sample pH (Extended Data Fig. 6d), in line with a previous study demonstrating pH dependence of prophage induction in *Escherichia coli* strains from the bladder^29^. Taken together, our analyses indicate that the virome is individual specific but similar between the stool and intestines of the same individual, and that the intestinal environment favours prophage induction, highlighting the importance of in situ sampling for capturing phage dynamics.

## Spatial variation of the host proteome

**Fig. 4:**
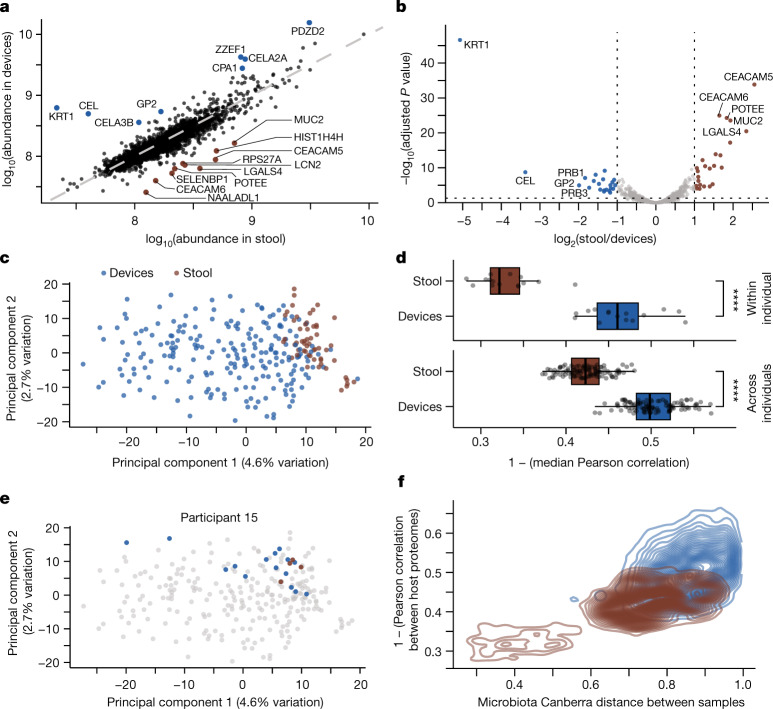
Human protein abundance differs between stool and intestinal samples. **a**, Median log_10_(abundance) of human proteins in stool samples (*n* = 56) compared with intestinal samples (*n* = 212). **b**, log_2_(fold change) of each protein abundance in stool relative to intestinal samples. A two-sample modified *t*-test with Benjamini–Hochberg correction was used. Proteins with absolute log_2_(fold change) > 1 and *P* < 0.05 are coloured on the basis of sample type and enrichment. **c**, PCA of normalized human protein abundance shows separation between intestinal and stool samples (*n* = 212 and 56, respectively). **d**, Human proteome composition varies significantly more between intestinal samples (*n* = 212) than between stool samples (*n* = 56), both within (top) and across (bottom) participants. Top, each circle is the median Pearson correlation coefficient of all sample pairs for a given participant. Bottom, each circle is the median of all correlation coefficients between all pairs of samples from any two participants (*n* = 105 for each intestinal and stool sample). ****P ≤ 0.0001, Bonferroni-corrected two-tailed Wilcoxon rank-sum test. **e**, PCA from **c** highlighting the clustering of intestinal and stool samples from participant 15 (*n* = 15 and 4, respectively). **f**, Canberra distance between microbiota compositions was higher in samples with less similar human proteomes for all sample pairs of a given type (*n* = 20,706 pairwise comparisons for devices, *n* = 1,485 pairwise comparisons for stool).

**Extended Data Fig. 7:**
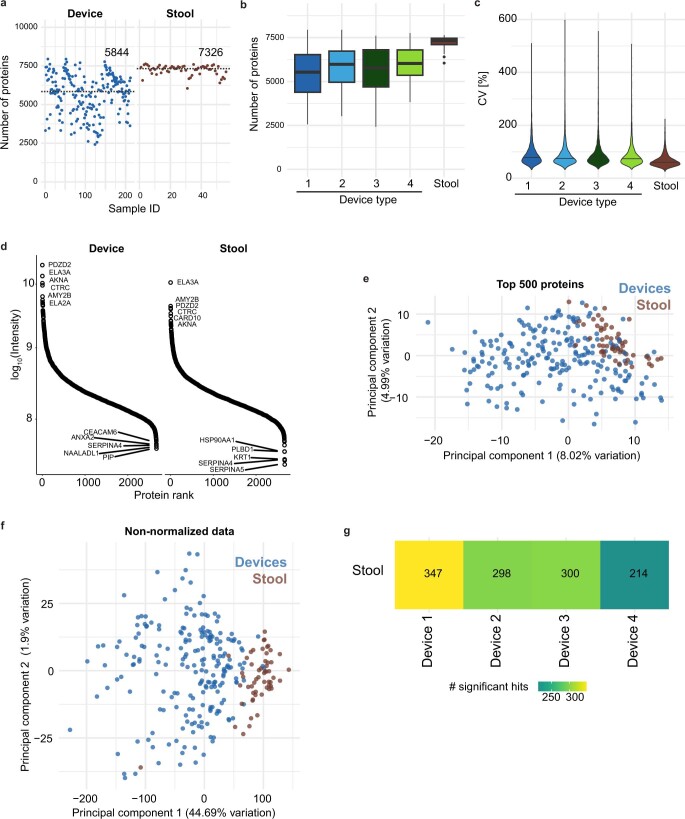
Comparison of the human proteome in intestinal versus stool samples. a) The number of host-associated proteins detected in each sample, colored by location (intestinal versus stool). Dotted line and number represent the mean. b) The number of host-associated proteins detected across samples from each device type and stool. Boxplot shows the median and 1^st^ and 3^rd^ quartiles. c) The distribution of coefficient of variation (CV) for all detected host proteins across device types and stool samples. d) Proteins were ranked based on mean log_10_(intensity) for intestinal and stool samples. The six most and five least abundant proteins are labelled for each location. e) A principal component analysis of the normalized abundance of the 500 most abundant host-associated proteins. Results are similar to Fig. 4c. Points are colored based on location of sample (intestinal versus stool). f) A principal component analysis of the non-normalized abundance of all host-associated proteins found in each sample. Results are qualitatively similar to Fig. 4c and e. Points are colored based on location of sample (intestinal versus stool). g) The number of host-associated proteins with significantly different abundance between stool and each device type based on a 5% false discovery rate (FDR). All panels used *n* = 56 stool and *n* = 212 device samples for analysis. In (b,c,g), *n* = 56 type 1, *n* = 54 type 2, *n* = 56 type 3, and *n* = 46 type 4 device samples were used.

A previous study in mice showed that host protein abundance depends strongly on location within the intestinal tract^3^, and our devices provide an unprecedented opportunity to quantify human host expression patterns in situ. We used liquid chromatography followed by tandem mass spectrometry (LC–MS/MS) to quantify human proteins in all intestinal and stool samples (Methods) and detected a comparable number to previous studies^30,31^ (Extended Data Fig. 7a), with a similar number of detected proteins (Extended Data Fig. 7b) and coefficient of variation in the abundance of detected proteins (Extended Data Fig. 7c) across device types. The most abundant proteins in stool samples (Extended Data Fig. 7d) were consistent with previous studies^31^. Filtering for proteins detected in 70% of samples, we detected and analysed 2,276 ± 269 human proteins per sample and observed significant differences in the abundance of some proteins between device samples and stool (Fig. 4a). A differential enrichment analysis identified sets of proteins that were indicators of regional specificity between the intestines and stool (Fig. 4b). We normalized abundance to the average across samples to account for the range of protein abundance and performed a principal component analysis (PCA). The human proteome clustered with similar qualitative features as the microbiota (Fig. 1e), with stool samples tightly clustering at high values of PC1 and intestinal samples displaying much greater variation along PC1 (Fig. 4c). Similar clustering was observed when considering only the 500 most abundant proteins (Extended Data Fig. 7e) or without normalization (Extended Data Fig. 7f). Moreover, the number of proteins with significantly different abundance between stool and each of the device types was highest between stool and type 1 devices and lowest between stool and type 4 devices (Extended Data Fig. 7g), probably reflecting longitudinal variation of the host proteome.

On the basis of Pearson correlation coefficients, the host proteome in stool samples was more variable across individuals than within individuals (Fig. 4d). In intestinal samples, the host proteome was similarly variable across individuals as within individuals and was more variable than in stool samples (Fig. 4d), reflecting broad separation from stool sample proteomes (Fig. 4c). Nonetheless, in some cases (for example, in participant 15), the host proteome of intestinal samples clustered tightly with that of stool samples (Fig. 4e), similar to the microbiota-based clustering of samples from participant 15 (Fig. 1e).

To determine whether variation in the host proteome was globally related to the variation in microbiota composition across samples (Fig. 1e), we compared the Pearson correlation coefficient of the host proteome with the Canberra distance between the microbiota composition of pairs of samples. Sample pairs with more correlated proteomes had more closely related microbiota (Fig. 4f).

Thus, the host proteome determined from stool is not representative of the host proteome in the intestines, which is globally correlated with microbiota composition in the intestines.

## Bile acid profiles along the intestinal tract

**Fig. 5:**
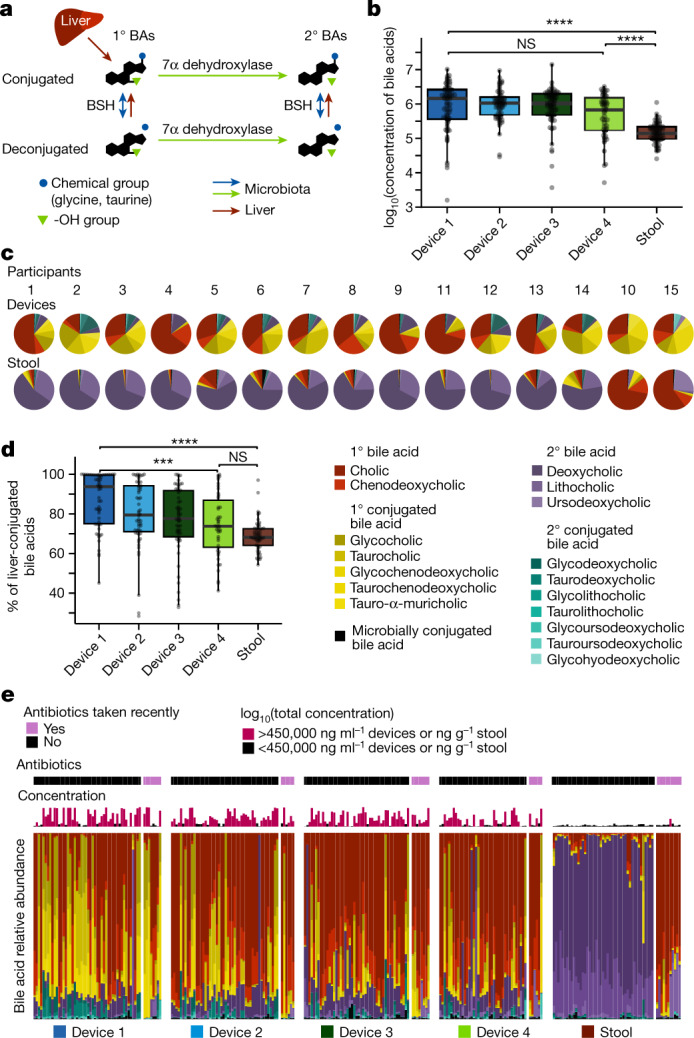
Devices capture different bile acid profiles along the intestinal tract compared with stool. **a**, Schematic of bile acid (BA) modifications by the liver and microbiota. The liver releases bile acids conjugated with glycine or taurine. Dehydroxylation by gut microorganisms converts primary (1°) to secondary (2°) bile acids. Microbial BSHs deconjugate amino acids from bile salts. **b**, The total concentration of all bile acids decreases along the intestinal tract (device type 1 to stool, *P* = 2.0 × 10^−9^; device type 4 to stool, *P* = 5.6 × 10^−4^; device type 1 to 4, *P* = 0.18). Shown are log_10_-transformed concentrations for intestinal (*n* = 58, 56, 57 and 47 for device types 1–4, respectively) or stool (*n* = 57) samples. **c**, The mean relative concentration of all bile acids for each participant in devices and stool. In all but two participants (10 and 15), DCA and LCA dominated the stool, but not the intestines. **d**, The percentage of liver-conjugated bile acids decreases significantly along the intestinal tract (device type 1 to stool, *P* = 2.2 × 10^−10^; device type 4 to stool, *P* = 0.20; device type 1 to 4, *P* = 3.2 × 10^−4^; *n* = 58, 56, 57 and 47 for device types 1–4, respectively, and *n* = 57 for stool samples). **e**, Relative abundance of bile acids for each sample arranged by device type. Participants are ordered 1–9, 11–14, 10, 15 within each device type. Antibiotic usage and log_10_(total concentration of bile acids) in the sample are also shown. Boxplots show the median and first and third quartiles. ***P ≤ 0.001, ****P ≤ 0.0001, Bonferroni-corrected two-sided Wilcoxon rank-sum test. Concentrations are in units of ng ml^–1^ or ng g^–1^ for devices and stool, respectively.

Bile acids are major chemical components of the human intestinal tract and are critical for food digestion, lipid absorption, host signalling and neurohormonal regulation of diverse physiological processes^5^. Bile acids have been implicated in a wide range of disorders, including inflammatory bowel disease (IBD)^32^, metabolic disorders^32^ and neurological diseases^33,34^. Glycine- and taurine-conjugated forms of the primary bile acids cholic acid (CA) and chenodeoxycholic acid (CDCA) are secreted from the liver and gallbladder into the duodenum and are then subjected to various microbial transformations (Fig. 5a)^4,35^. Approximately 95% of bile acids that reach the distal ileum are transported through the epithelium into the portal vein and return to the liver^4^, where they are transformed back into bile salts and re-secreted, creating the potential for longitudinal bile acid gradients along the intestinal tract. To quantify bile acid profiles along the intestinal tract, we performed targeted LC–MS/MS metabolomics with multiple-reaction monitoring (MRM) on 17 commonly investigated bile acids in the supernatants of all intestinal and stool samples. The total concentrations of bile acids and their relative levels in intestinal samples were highly variable (Fig. 5b), yet distinct trends were observed. The total concentration of bile acids was generally decreased by ~2-fold in samples collected by type 4 devices and ~10-fold in stool relative to samples collected by type 1 devices (Fig. 5b), probably reflecting active reabsorption of bile acids along the intestines^4^.

In contrast to all other participants, the stool bile acid profiles of two participants (10 and 15) were similar to their intestinal samples in that they contained a dominant fraction of CA (Fig. 5c). These are the two participants who reported recent antibiotic use and had substantially different microbiota composition to the other participants (Fig. 1e). The intestinal and stool samples from participants 10 and 15 also lacked deoxycholic acid (DCA) and lithocholic acid (LCA) (Fig. 5c), suggesting that the microorganisms necessary for the 7*α*-dehydroxylation reaction required to produce these bile acids may have been eliminated by the antibiotics.

In all other participants, the relative levels and dominant bile acid classes differed markedly between intestinal and stool samples. Intestinal samples were mostly dominated by the primary bile acid CA, whereas stool samples were dominated by the secondary bile acid DCA (Fig. 5c), probably owing to prolonged exposure of bile acids to microbial enzymes in the colon. These results highlight that stool-based measurements do not reflect the true composition of bile acids along the intestinal tract.

## Gradients of bile acid modifications

**Extended Data Fig. 8:**
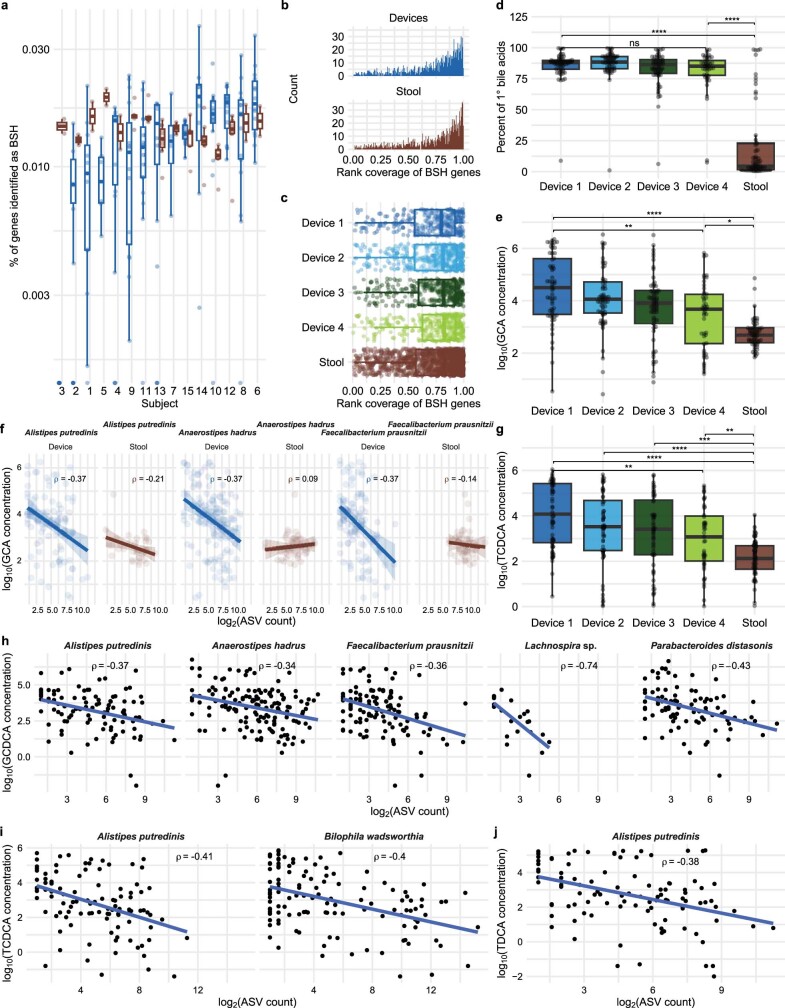
Microbial bile salt hydrolase genes exhibited similar abundance and diversity in intestinal and stool samples despite differences in conjugated bile acids along the intestinal tract. a) Open reading frames identified as bile salt (cholylglycine) hydrolase (BSH) enzymes via a hidden Markov model (HMM) search, normalized by the total number of open reading frames detected in the sample. b) The distribution of rank coverage of *bsh* genes was similar between intestinal and stool samples. c) Rank coverages of *bsh* genes in devices of each type and in stool are similar. (all *P* > 0.90). d) Percentage of primary (hydroxylated) bile acids was similar across device types and was lower in stool compared with intestinal samples (top to bottom: *P* = 3.7 × 10^−13^, 1.3 × 10^−10^, and 0.91). e) Glycocholic acid (GCA) concentration decreased along the intestinal tract (top to bottom: *P* = 1.6 × 10^−12^, 0.035, and 0.003). f) The log_2_(ASV count) of *Alistipes putredinis*, *Anaerostipes hadrus*, and *Bilophila wadsworthia* was negatively correlated (Spearman; *P* = 0.0068, 0.0004, and 0.0068 in devices and *P* = 0.63, 0.84, and 0.70 in stool, respectively) with log_10_(GCA concentration). Only ASVs with *P* < 0.01 after a Benjamini-Hochberg correction in device samples are shown. g) Taurochenodeoxycholic acid (TCDCA) concentration decreased along the intestinal tract (top to bottom: *P* = 0.0070, 1.9 × 10^−4^, 4.2 × 10^−6^, 1.5 × 10^−10^, and 0.0020). h) log_2_(ASV read count) of *Alistipes putredinis* and *Bilophila wadsworthia* in devices was negatively correlated (Spearman; *P* = 2.5 × 10^−5^ and 3.3 × 10^−7^, respectively) with log_10_(TCDCA concentration). Only ASVs with *P* < 0.01 after a Benjamini-Hochberg correction in device samples are shown. i) log_2_(ASV read count) of *Bilophila wadsworthia* in devices was negatively correlated (Spearman; *P* = 2.4 × 10^−5^) with log_10_(concentration of taurodeoxycholic acid (TDCA)). Only ASVs with *P* < 0.01 after a Benjamini-Hochberg correction in device samples are shown. In (a–c), *n* = 175 device and *n* = 58 stool samples were used for analysis. In (d–h), *n* = 210 device samples and *n* = 56 stool samples were used for analysis. All boxplots show the median and 1^st^ and 3^rd^ quartiles. ns: not significant, ****: *P* ≤ 0.0001, Bonferroni-corrected two-sided Wilcoxon rank-sum test. Bile acids shown are log_10_-transformed concentrations in units of ng/mL or ng/g for intestinal or stool samples, respectively.

Bile acids are modified in the intestinal tract by microbial enzymes that deconjugate glycine or taurine or remove hydroxyl group(s) from the steroid backbone (Fig. 5a). Deconjugation is performed by bile salt hydrolases (BSHs), which cleave glycine and taurine from the bile acid backbone. BSH homologues are present in ~25% of bacterial strains sequenced from human stool samples^36^. Although there was only a small (albeit significant; *P* = 0.03, Wilcoxon rank-sum test) difference in the abundance of BSH genes between intestinal and stool samples (Extended Data Fig.  8a) and little variation in rank coverage between intestinal and stool samples (Extended Data Fig. 8b) or among device types based on metagenomic sequencing (Extended Data Fig. 8c), we observed a significant monotonic decrease in the percentage of liver-conjugated bile acids in samples from device type 1 to device type 4 (Fig. 5d), reflecting a trend of deconjugation along the intestinal tract and into stool^37^.

Dehydroxylation reactions require several enzymes to transform primary to secondary bile acids and are thought to occur predominantly in the low-redox state of the colon^37^. In line with the majority of dehydroxylation occurring in the large intestine, we found that secondary bile acids did not change substantially across device types but were significantly increased in stool samples, which were dominated by secondary unconjugated bile acids (Fig. 5c and Extended Data Fig. 8d). The presence of secondary bile acids in intestinal samples is probably due to dehydroxylation of primary bile acids in the small intestine or re-introduction of secondary bile acids present in bile into the duodenum; secondary bile acids are expected to be in bile given previous evidence that they represent ~25% of the bile acids secreted from the gallbladder^37^. In sum, the variation in bile acid profiles that we detected throughout the intestinal tract (Fig. 5e) demonstrates regionality of the microbial activity and biochemical environment of the intestines, further highlighting the limitations of relying on stool for microbiome and bile acid studies.

## Microbial links to bile acid deconjugation

We sought to exploit the variation in conjugated bile acid concentrations across intestinal samples to identify candidate bacterial species responsible for deconjugation. Given the monotonic decrease in the fraction of liver-conjugated bile acids from device type 1 to 4 (Fig. 5d), we reasoned that the abundance of the microbial taxa most responsible for deconjugation might be inversely correlated with the concentration of conjugated bile acids, even against the background of potential regulation of deconjugation by the host or antimicrobial activity of bile acids.

**Fig. 6:**
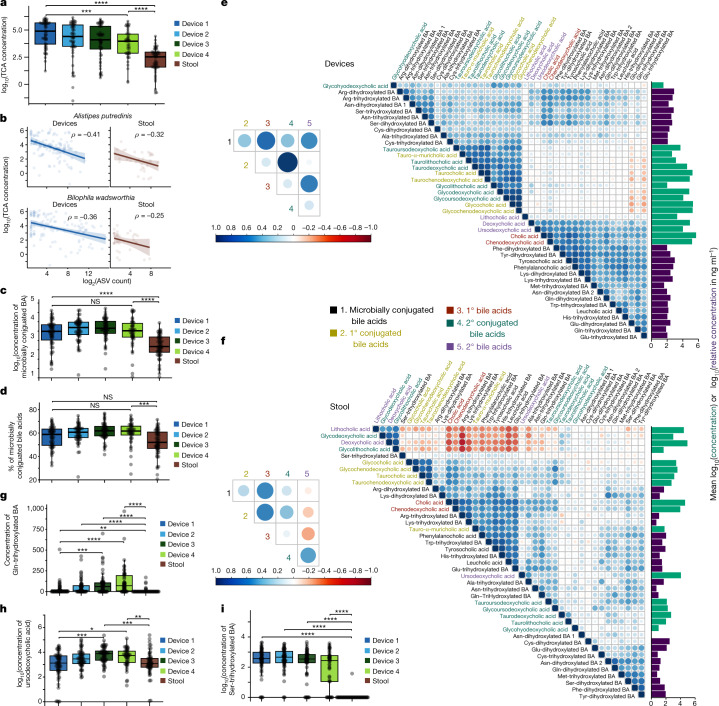
Bile acid relationships in intestines and stool. **a**, TCA concentration decreases along the intestinal tract. Shown are log_10_-transformed concentrations. Device type 1 to 4, *P* = 10^−3^; device type 1 to stool, *P* = 6.2 × 10^−14^; device type 4 to stool, *P* = 5.5 × 10^−7^. **b**, log_2_(read count) of an *A.* *putredinis* ASV and a *B.* *wadsworthia* ASV was negatively correlated (*P* = 0.0020 and *P* = 0.0042, respectively) with TCA concentration. Correlations were weaker in stool samples (*P* = 0.36 and *P* = 0.56, respectively). Correlations are Spearman correlations with Benjamini–Hochberg correction. Only ASVs with *P* < 0.01 after correction in devices are shown. Points are individual intestinal (*n* = 210) or stool (*n* = 56) samples for which both 16S rRNA sequencing and metabolomics data were available. **c**, The concentration of microbially conjugated bile acids is significantly higher in intestinal samples than in stool samples. The concentration did not differ significantly across device types. Device type 1 to 4, *P* = 1; device type 1 to stool, *P* = 3.5 × 10^−6^; device type 4 to stool, *P* = 1.6 × 10^−6^. **d**, The percentage of microbially conjugated bile acids increases along the intestinal tract and was significantly higher in intestinal samples than in stool. Device type 1 to 4, *P* = 0.40; device type 1 to stool, *P* = 0.23; device type 4 to stool, *P* = 8.0 × 10^−4^. **e**,**f**, Correlations between bile acid profiles differ between intestinal (**e**) and stool (**f**) samples. Shown are Pearson correlation coefficients using log_10_-transformed concentrations. Horizontal bars show the mean absolute concentration (green) or relative concentration (purple) (Methods). Bile acid ordering was determined by hierarchical clustering. Insets show the Pearson correlation coefficient for aggregated classes. **g**–**i**, Concentration of the respective bile acid across devices and stool. *P* values from top to bottom for **g**: 4.3 × 10^−9^, 7.5 × 10^−9^, 5.1 × 10^−7^, 7.0 × 10^−3^, 1.8 × 10^−4^ and 6.5 × 10^−4^. *P* values from top to bottom for **h**: 0.001, 5.8 × 10^−5^, 0.023 and 1.4 × 10^−4^. *P* values from top to bottom for **i**: 5.9 × 10^−13^, 1.5 × 10^−16^, 8.5 × 10^−20^ and 6.2 × 10^−19^. All boxplots show the median and first and third quartiles. Points are individual intestinal (*n* = 58, 56, 57 and 47 for device types 1–4, respectively) or stool (*n* = 57) samples, unless otherwise indicated. Concentrations in ng ml^–1^ (intestinal) or ng g^–1^ (stool). *P ≤ 0.1, **P ≤ 0.01, ***P ≤ 0.001, ****P ≤ 0.0001, Bonferroni-corrected two-sided Wilcoxon rank-sum test.

We focused on primary bile acids, which dominate the pool of conjugated bile acids, namely glycocholic acid (GCA) and taurocholic acid (TCA). Previous studies have shown that diet can influence bile acid profiles in mice^25^, motivating examination of whether certain types of food consumed during our study affected CA, GCA or TCA concentration in the human intestinal tract. The concentration of these bile acids was not significantly affected by diet, but participants who consumed vegetables during the study had a significantly higher ratio of TCA to GCA concentration (*P* = 0.002, Bonferroni-corrected Wilcoxon rank-sum test), and participants who had consumed dairy had a significantly higher ratio of GCA to TCA concentration (*P* = 0.026, Bonferroni-corrected Wilcoxon rank-sum test). A previous study linked milk-derived fat to TCA production in the gallbladder and *B.* *wadsworthia* expansion in the stool of mice^25^, motivating investigation of the links between deconjugation and microbial taxa along the intestinal tract. The concentration of both GCA and TCA decreased from device type 1 to 4 and was significantly lower in stool (Fig. 6a and Extended Data Fig. 8e). GCA concentration was negatively correlated with the log_2_(abundance) of *Anaerostipes hadrus* and *Faecalibacterium prausnitzii* (Extended Data Fig. 8f), and TCA concentration was negatively correlated with the log_2_(abundance) of *Alistipes putredinis* and *B.* *wadsworthia* (Fig. 6b). Across all participants, we analysed our 440 high-quality MAGs (completeness of >90% and contamination of <10%, dereplicated to 99% average nucleotide identity (ANI)) and searched for the canonical BSH gene in each using a hidden Markov model. We found putative BSH genes in *A.* *hadrus* (7 of 8 MAGs) and *A.* *putredinis* (4 of 4 MAGs), in accordance with previous literature^38^. By contrast, none of the 12 *F.* *prausnitzii* MAGs nor the 3 *B.* *wadsworthia* MAGs contained any putative BSH genes, suggesting that these taxa may use glycine and taurine^25^ generated by other microbial deconjugation reactions.

A number of negative correlations (implying potential microbial deconjugation) involving other taurine- and glycine-conjugated bile acids were observed (Extended Data Fig. 8g–j). Taurochenodeoxycholic acid (TCDCA) concentration (Extended Data Fig. 8g) was also negatively correlated with *B.* *wadsworthia* and *A.* *putredinis* log_2_(abundance) (Extended Data Fig. 8i), and taurodeoxycholic acid (TDCA) was negatively correlated with *A. putredinis* log_2_(abundance) (Extended Data Fig. 8j), suggesting that these species interact with various taurine-conjugated bile acids. We focused mainly on *B.* *wadsworthia* because it was differentially abundant in intestinal samples compared with stool (Fig. 1f). The name of the *Bilophila* genus reflects its growth stimulation by high concentrations of bile^39^, and the higher ratio of GCA to TCA concentration in participants who consumed dairy is potentially due to the ability of *B.* *wadsworthia* to deconjugate TCA to use taurine for growth^25^. Notably, in stool, the relative abundance of *B.* *wadsworthia* and *A.* *putredinis* was correlated only weakly or not at all with TCA concentration (Fig. 6b), indicating that the devices identify correlations between bile acids and microorganisms that would not be evident from stool.

## Amino acid-specific bile acid conjugation

Bile acids conjugated to amino acids other than glycine and taurine (for example, tyrosocholic acid (TyroCA), leucocholic acid (LeuCA) and phenylalanocholic acid (PhenylCA)) were recently discovered in the gut of mice and stool of humans^35^. Synthesis of TyroCA, LeuCA and PhenylCA^35^ by microorganisms that reside in the intestinal tract has been reported in vitro^40^, and the levels of these conjugates differ significantly between healthy and disease states such as Crohn’s and IBD^35,41^. Furthermore, these microbially conjugated bile acids are agonists of the human farnesoid X receptor^20^. Despite widespread interest in these conjugates, very few studies have measured their levels, particularly in host-relevant contexts such as the intestines, where longitudinal trends are completely unknown. Using untargeted LC–MS/MS analysis with data-dependent MS/MS acquisition, we detected 22 microbially conjugated bile acids in various hydroxylation forms across 13 amino acids in the intestinal samples of all participants (Supplementary Table 6). Microbially conjugated bile acids were at significantly higher concentrations (Fig. 6c) and accounted for a significantly higher fraction of the bile acid pool (Fig. 6d) in intestinal samples compared with stool.

The concentrations of primary and secondary liver-conjugated bile acids were highly correlated, while the total concentration of microbially conjugated bile acids was correlated with that of deconjugated bile acids across intestinal samples (Fig. 6e). In stool, the total concentration of microbially conjugated bile acids was correlated with the concentration of primary deconjugated bile acids and inversely correlated with the concentration of secondary deconjugated bile acids (Fig. 6f). These findings emphasize the effect of different anatomical regions and routes of formation and degradation on liver-conjugated bile acids (glycine and taurine conjugates) and microbially conjugated bile acids, further highlighting major differences in the metabolite environment of the intestines versus stool.

Across all intestinal samples in this study, the 22 microbially conjugated bile acids clustered into two groups: the concentration of cysteine-, serine- and alanine-conjugated bile acids exhibited strong correlation with the concentration of liver-conjugated bile acids such as GCA and TCA, while the concentration of glutamic acid-, glutamine-, tryptophan-, leucine-, arginine-, phenylalanine-, lysine- and tyrosine-conjugated bile acids correlated strongly with the concentration of unconjugated bile acids such as CA and CDCA (Fig. 6e). The clustering of correlation profiles of many di- and trihydroxylated bile acids of the same amino acid underscores the amino acid dependence of trends in microbially conjugated bile acids. This clustering was not present in stool samples (Fig. 6f); instead, both liver-conjugated and unconjugated bile acids correlated with various bile acid types conjugated with a given amino acid, which were sometimes largely uncorrelated with each other (for example, glutamate dihydroxlated and glutamate trihydroxylated; Fig. 6f).

Of the 22 microbially conjugated bile acids detected in intestinal samples, 20 were reliably detected in stool despite lower overall levels of bile acids in the stool (Fig. 6e,f). Even with variation in concentration, several microbially conjugated bile acids exhibited a gradient across device types and showed amino acid-specific trends. Glutamine-conjugated bile acids increased from type 1 to 4 devices (Fig. 6g) similarly to unconjugated secondary bile acids such as ursodeoxycholic acid (Fig. 6h), in line with the hypothesis that microbial conjugation along the small intestine causes some bile acids to increase in concentration. However, serine-conjugated bile acids decreased from type 1 to 4 devices (Fig. 6i), similar to trends in liver-conjugated bile acids such as TCDCA (Extended Data Fig. 8g); this decrease in concentration is probably due to flow of microbially conjugated bile acids through enterohepatic circulation and deconjugation along the intestines when they are excreted in bile. Microbial deconjugation is the most parsimonious explanation for the decreases in concentration of certain microbially conjugated bile acids between device types 1 and 4 (Fig. 6c,d). Although PhenylCA, LeuCA and TyroCA are microbially conjugated, a previous study reported that PhenylCA, LeuCA and TyroCA are not deconjugated by intestinal microbiota^35^; we found that PhenylCA, LeuCA and TyroCA were not among the bile acids that decreased from device type 1 to 4. Together, these observations indicate that some microbially conjugated bile acids may be deconjugated by microorganisms while others are not. Previous studies did not detect these opposing longitudinal trends in microbially conjugated bile acids^41^. These data represent a spatial investigation of microbially conjugated bile acids in the human intestines and identify trends that are amino acid specific.

## Discussion

Thus far, studies of the human gut microbiome and metabolites have relied mainly on stool. In this study, enabled by the development and implementation of an ingestible sampling device, we demonstrated that analysis of stool provides neither a complete nor an accurate representation of the longitudinal and temporal variability of the microbiota composition, virus activity, host proteome and bile acid contents within the intestines. The trends in microbially conjugated bile acids were strong and novel, and, although it remains unclear why bile acids exhibit distinct abundance profiles along the intestinal tract, our data provide the opportunity to identify the bacterial species and genes responsible for these transformations and profiles. The wide variability among intestinal samples, both within and across individuals, highlights the dynamic environment of the small intestine and the need for increased sampling (both longer term and in larger cohorts) to determine baseline variation expected in healthy individuals before studies of disease states can be robustly evaluated for differences in spatiotemporal variability or overall community composition. With that understanding, we envision interrogating how diet and disease differentially influence the intestinal microbiota, metabolome, virome and proteome. Indeed, measurements from the proximal intestinal microbial ecosystem will be critical for future clinical studies of spatially restricted human intestinal diseases and therapeutic interventions directed at these disorders.

In a companion study^19^, we interrogate spatial and temporal differences in intestinal metabolomes in further detail, including changes to dietary and lipid compounds. We report the detection in humans of sulfonolipids, which were associated with several microbial taxa, as well as an association of FAHFA lipids with *Blautia* species. Taken together, these studies demonstrate the feasibility and utility of a safe and non-invasive method for collection, characterization and quantification of the intestinal microbiota, metabolome, host proteins and bile acids along the human intestinal tract during normal digestion. This new capability, when deployed at scale, should improve understanding of the dynamic and intertwined nature of human metabolic pathways with our resident gut microorganisms and their potential involvement in normal physiology and disease.

## Methods

### Ingestible capsule sampling device

The capsule sampling device (CapScan, Envivo Bio) consists of a hollow elastic collection bladder capped by a one-way valve. The device is prepared for packaging by evacuating the collection bladder, folding it in half and packaging the folded device inside a dissolvable capsule measuring 6.5 mm in diameter and 23 mm in length, onto which an enteric coating is applied. The capsule and the enteric coating prevent contamination of the collection bladder from oral–pharyngeal and gastric microorganisms during ingestion. When the device reaches the target pH, the enteric coating and capsule disintegrate. The target pH is 5.5 for type 1, 6 for type 2 and 7.5 for type 3 and type 4, with type 4 also having a time delay coating to bias collection towards the ascending colon. After the enteric coating disintegrates, the collection bladder unfolds and expands into a tube 6 mm in diameter and 33 mm in length, thereby drawing in up to 400 µl of gut luminal contents through the one-way valve. The one-way valve maintains the integrity of the sample collected inside the collection bladder as the device moves through the colon and is exposed to stool.

In this study, participants concurrently ingested sets of four devices, each with distinct coatings to target the proximal to medial regions of the small intestine (coating types 1 and 2) and more distal regions (coating types 3 and 4). After sampling, the devices were passed in the stool into specimen-collection containers and immediately frozen. After completion of sampling, the stool was thawed and the devices were retrieved by study staff. The elastic collection bladders were rinsed in 70% isopropyl alcohol and punctured with a sterile hypodermic needle attached to a 1-ml syringe for sample removal. Samples were transferred into microcentrifuge tubes, and the pH was measured with an InLab Ultra Micro ISM pH probe (Mettler Toledo). A 40-µl aliquot was spun down for 3 min at 10,000 RCF, and its supernatant was used for metabolomics analysis while the pellet was used for proteomics analysis. The rest of the sample was frozen until being thawed for DNA extraction.

### Study design

The study was approved by the WIRB-Copernicus Group institutional review board (study 1186513), and informed consent was obtained from each participant. Healthy volunteers were selected to exclude participants with clinically detectable gastrointestinal conditions or diseases that would potentially interfere with data acquisition and interpretation. There was no blinding, randomization, or statistical methods to determine sample size.

Participants met all of the following criteria for study inclusion: (1) age between 18 and 70 years; (2) American Society of Anesthesiologists (ASA) physical status class risk of 1 or 2; (3) for women of childbearing potential, a negative urine pregnancy test within 7 days of the screening visit and willingness to use contraception during the entire study period; and (4) fluency in English, with an understanding of the study protocol and ability to supply informed written consent, as well as compliance with study requirements.

Individuals with any of the following conditions or characteristics were excluded from the study: (1) a history of any of the following: prior gastric or oesophageal surgery, including lap banding or bariatric surgery, bowel obstruction, gastric outlet obstruction, diverticulitis, IBD, ileostomy or colostomy, gastric or oesophageal cancer, achalasia, oesophageal diverticulum, active dysphagia or odynophagia, or active medication use for any gastrointestinal conditions; (2) pregnancy or planned pregnancy within 30 days of the screening visit or breast-feeding; (3) any form of active substance abuse or dependence (including drug or alcohol abuse), any unstable medical or psychiatric disorder, or any chronic condition that might, in the opinion of the investigator, interfere with conduct of the study; or (4) a clinical condition that, in the judgment of the investigator, could potentially pose a health risk to the individual while they were involved in the study.

Fifteen healthy individuals were enrolled in this study, and each swallowed at least 17 devices over the course of 3 days (for demographics, see Supplementary Table 1). Daily instructions included the following guidelines: record all foods and the time they were consumed throughout the day; if you work out, do so in the morning; eat breakfast and lunch as usual; swallow a set of four devices 3 h after lunch with up to two-thirds cup water; do not eat or drink anything for at least 2 h after swallowing the devices; if hungry after 2 h, snack lightly (up to 200 calories); do not drink any caffeinated beverages after lunch until the next morning; collect all stool starting 6 h after swallowing this set of devices until 48 h after swallowing the next set of devices; eat dinner as usual at least 6 h after lunch; swallow a set of four devices 3 h after dinner with two-thirds cup water; after swallowing this set, do not eat or drink anything until the morning. Alcohol consumption and diet contents were not restricted. All ingested devices were recovered, and no adverse events were reported during the study. Of the 255 ingested devices, 15 were set 1 safety devices (not included in analysis) and 22 contained gas or low sample volume. Saliva samples were collected after evening meals and immediately frozen at –20 °C. A sample of every bowel movement during the study was immediately frozen by the participant at −20 °C. A total of 306 samples (*n *= 29 saliva, *n *= 218 devices, *n *= 59 stool) provided sufficient material for multi-omic analyses (Extended Data Fig. 2a). Furthermore, participant 1 provided additional samples for assessment of replicability and microbial blooming.

### Blooming analysis

To assess the effect of in-body incubation on the contents of the devices between the time of sample collection and sample retrieval, a set of four devices (one of each type) was ingested by participant 1. Following exit in a bowel movement at 32 h, the devices were immediately transferred to an anaerobic chamber and incubated at 37 °C. An aliquot of each sample was taken at 32 h (immediately after the bowel movement), 58 h and 87 h (with the latter two time points simulating lengthier gut transit times). These aliquots were subjected to 16S rRNA gene amplicon sequencing. The rank abundance of the 30 most abundant ASVs at 32 h shifted at 58 h by a median of 8–16 ranks and at 87 h by 12–30 ranks (Extended Data Fig. 1). The 9–17 ASVs that increased from below to above the detection limit during incubation collectively accounted for a relative abundance of 9.4–13.8% after 58 h and 5.2–18% after 87 h, presumably because of growth during incubation. Thus, although outgrowth can potentially alter assessments of microbiota composition, major changes are not expected for transit times of ~58 h or less.

### Time-lapse imaging

Agarose (1%) pads with BHI medium were sealed using VALAP (1:1:1 Vaseline:lanolin:paraffin) and maintained at 37 °C using a heated environmental chamber (HaisonTech). Phase-contrast images were collected on a Nikon Ti-E epifluorescence microscope using µManager (v.1.4)^42^.

### DNA extraction and 16S rRNA gene sequence analysis

Of the 240 devices, 218 collected >50 µl of intestinal fluids and were subjected to DNA extraction and 16S rRNA gene and metagenomic sequencing; the remainder sampled <50 µl or were filled with gas, most likely from the colon.

For the 218 devices that sampled >50 µl, DNA was extracted using a Microbial DNA extraction kit (Qiagen)^43^ and 50 µl from a device, 200 µl of saliva or 100 mg of stool.

16S rRNA amplicons were generated using Earth Microbiome Project-recommended 515F/806R primer pairs and 5PRIME HotMasterMix (Quantabio, cat. no. 2200410) with the following programme in a thermocycler: 94 °C for 3 min; 35 cycles of 94 °C for 45 s, 50 °C for 60 s and 72 °C for 90 s; and 72 °C for 10 min. PCR products were cleaned, quantified and pooled using the UltraClean 96 PCR Cleanup kit (Qiagen, cat. no. 12596-4) and Quant-iT dsDNA High-Sensitivity Assay kit (Invitrogen, cat. no. Q33120). Samples were sequenced with 250-bp reads on a MiSeq instrument (Illumina).

Sequence data were demultiplexed using the Illumina bcl2fastq algorithm at the Chan Zuckerberg Biohub Sequencing facility. Subsequent processing was performed with the R statistical computing environment (v.4.0.3)^44^ and DADA2 as previously described^43^ using pseudo-pooling^45^. truncLenF and truncLenR parameters were set to 250 and 180, respectively. Taxonomy was assigned using the Silva rRNA database (v.132)^46^. Samples with >2,500 reads were retained for analyses. We obtained sufficient sequencing reads from 210 samples, which were the focus of subsequent analyses, along with sequencing data from 29 saliva and 58 stool samples (one participant provided only one saliva sample, and one stool sample had insufficient sequencing reads; Extended Data Fig. 2a).

A phylogenetic tree was constructed using phangorn as previously described^47^. Shannon diversity was calculated using the phyloseq::estimate_richness function, which is a wrapper for the vegan::diversity function^48,49^. Because intestinal samples were often dominated by a single ASV (Fig. 2c), the Canberra distance metric was used for pairwise beta-diversity comparisons. Only the 455 ASVs represented by ≥3 reads in ≥5% of samples were used to calculate distances.

### Limitations and contamination analysis

One limitation of our study is that the exact location of sample collection within the intestines could not be clearly defined or validated. Variability in intestinal peristalsis and pH during normal digestion may cause devices within a set to experience different pH gradients; hence, they may open before or after their intended collection sites. Despite this limitation, analysis of 210 intestinal samples from 15 individuals showed consistent trends of biochemical and microbial activity in the human intestines. More consistent sampling along a longitudinal gradient might be attained in future studies by collecting multiple sequential samples into a single device in a timed manner.

The significantly different bile acid profiles in intestinal compared with stool samples indicate that it is unlikely that stool contaminated the intestinal sampling devices during transit or sample recovery. However, because of the large increase in microbial density along the intestinal tract^37^, even a minute amount of stool contamination could affect microbiota composition. We therefore used a statistical approach to identify samples as potentially contaminated on the basis of microbial community composition. Given the directional motility of the intestinal tract, one would expect intrinsic overlap between intestinal and stool microbial communities. Latent Dirichlet allocation with the topicmodels R package^50^ was used to identify co-occurring groups of microorganisms (‘topics’^51^) from intestinal and stool samples for each participant. For each intestinal sample, the cumulative probability of topics identified as derived from the same participant’s stool was computed. Device samples with ≥10% of the total community identified as potentially originating from stool topics were flagged as possibly contaminated. Using this very conservative definition, 38 of the 210 intestinal samples with adequate sequencing read counts (originating from 12 of the study participants) were identified as possibly contaminated. All analyses presented in this study used all available data to avoid bias, but re-analysis of all data after removing the 38 samples that showed any signal of potential contamination from stool resulted in the same statistical trends as with the complete group of samples.

### Metagenomic sequencing

Extracted DNA from all samples was arrayed in a 384-well plate, and concentrations were normalized after quantification using the PicoGreen dsDNA Quantitation kit (ThermoFisher). DNA was added to a tagmentation reaction, incubated for 10 min at 55 °C and immediately neutralized. Mixtures were added to ten cycles of a PCR that appended Illumina primers and identification barcodes to allow for pooling of samples during sequencing. One microlitre of each well was pooled, and the pooled library was purified twice using AMPure XP beads to select appropriately sized bands. Finally, library concentration was quantified using a Qubit instrument (ThermoFisher). Sequencing was performed on a NovaSeq S4 instrument with read lengths of 2 × 146 bp.

### Preprocessing of raw sequencing reads and metagenomic assembly

Skewer (v.0.2.2)^52^ was used to remove Illumina adaptors, after which human reads were removed with Bowtie2 (v.2.4.1)^53^. Metagenomic reads from a single saliva, intestinal or stool sample were assembled with MEGAHIT (v.1.2.9)^54^. Assembled contigs were binned with MetaBAT 2 (v.2.15)^55^ into 7,655 genome bins. checkM (v.1.1.3)^56^ and quast (v.5.0.2)^57^ were used to assess quality; bins with >75% completeness and <25% contamination were dereplicated at 99% ANI (strain level) with dRep (v.3.0.0)^58^, resulting in 696 representative MAGs across all samples. GTDB-Tk was used to assign taxonomy^59^. Default parameters were used for all computational tools.

### Strain isolation from intestinal and stool samples

Isolates were obtained directly from samples or from communities derived from passaging of samples^60^ by either plating or fluorescence-activated cell sorting (FACS)^61^. For plating, samples were serially diluted tenfold onto BHI + 10% defibrinated horse blood (BHI-blood) plates and incubated for 72 h at 37 °C in an anaerobic chamber. Single colonies were re-streaked onto BHI-blood plates. This process was repeated an additional two times to ensure that the colony was axenic. Single colonies were then picked into a 2-ml deep-well plate containing 500 µl of BHI supplemented with menadione (vitamin K), cysteine and hemin (BHIS). In certain cases, Reinforced Clostridial Medium supplemented with menadione (vitamin K), cysteine and hemin (RCMS) was used instead. For FACS, single cells were sorted into BHIS using a previously described protocol that allows for isolation of strict anaerobes^61^.

After 72 h of growth in an anaerobic chamber at 37 °C, frozen stocks of all isolates were made using a final concentration of 12% glycerol. To identify isolates, cultures were spun down and pellets were resuspended with PCR-grade water in a 1:1 ratio. The primers 5′-AGAGTTTGATCCTGGCTCAG-3′ and 5′-GACGGGCGGTGWGTRCA-3′ were used to amplify the 16S rRNA gene. The PCR product was sent for Sanger sequencing, and sequences were filtered using sangeranalyseR with default parameters^62^. These sequences were searched against the rRNA/ITS BLAST database, and the top species hit was used to identify the strain.

### Analysis of CAZyme and AMR content

Putative genes were called on assembled contigs for each sample or on assembled MAGs using Prodigal^63^. CAZyme genes were identified using run_dbcan.py (v.3.0.5)^64^ with default parameters (searching with HMMER, eCAMI and DIAMOND). Genes identified in at least two of three programmes were dereplicated to create a curated database. Metagenomic reads for each sample were mapped against this database to calculate the percentage of reads mapped. AMR genes were identified using rgi (v.5.2.0)^28^ with default parameters. All identified genes were filtered for >90% coverage and dereplicated to create a curated database of AMR genes. Metagenomic reads for each sample were mapped against this database to calculate the percentage of reads mapped.

CARD is known to be biased towards pathogens such as *Escherichia*/*Shigella* species^28^, and indeed the relative abundance of *Escherichia*/*Shigella* species was highly positively correlated with the abundance of AMR genes in intestinal samples (Extended Data Fig. 5h). In stool samples, although no ASVs were positively correlated with the percentage of reads aligned to CARD, the abundance of the Enterobacteriaceae family was positively correlated, as was that of the Bacteroidaceae family (Extended Data Fig. 5i). To determine whether this correlation was driven by efflux activity, we recomputed AMR gene abundance while ignoring the 1,273 genes annotated as efflux pumps. In this analysis, intestinal samples did not exhibit significantly higher numbers of reads mapping to non-efflux AMR genes (Extended Data Fig. 5j). We identified AMR genes in each of our MAGs and found that Enterobacteriaceae possessed ~10- to 100-fold more AMR genes (normalized to the total number of genes) than other taxonomic families (Extended Data Fig. 5k).

### Viral contig identification

After assembly, contigs >1 kb in length were analysed using VirSorter2 (ref. ^65^), DeepVirFinder^66^ and VIBRANT^67^. Contigs identified as viral by at least one algorithm (VirSorter2 score ≥0.9, or DeepVirFinder score ≥0.9 and *P* < 0.05, or VIBRANT score of medium quality or higher) were clustered using an ANI cut-off of 0.95 and coverage cut-off of 85%. The quality of the clustered contigs was analysed using CheckV^68^, which also classified viral contigs as prophages if they contained both viral and bacterial regions.

### Detection of prophage induction events

The algorithm PropagAtE^69^ was used to identify active prophages with default parameters. In each sample, the total reads were first rarefied so that the number of reads mapped as viral was 5 × 10^5^ (six device samples and ten saliva samples had fewer than 5 × 10^5^ reads, and hence all reads from these samples were used for analyses). The reads were then mapped to the prophage contigs with a minimum per-cent identity of 97%. The algorithm identifies a prophage as active (induced) when the ratio of prophage to host depth for that contig is >2 and the prophage region has >50% coverage.

### Proteomics sample preparation

After thawing samples, 20 µl of MS-grade water (Pierce) was added to each sample and the mixture was vortexed. Twenty microlitres of this mixture was transferred to a 96-well plate (AFA-TUBE TPX plate, cat. no. 520291, Covaris). Twenty microlitres of cell lysis buffer (containing Tris, CAA, TCEP and 8% SDS)^9^ was added to each sample aliquot, and samples were boiled for 10 min in a PCR thermocycler (Eppendorf) to achieve reduction of disulfide bridges and alkylation of cysteines and to boost protein denaturation. Following boiling, samples were placed in a −80 °C freezer to ensure microbial capsule dissociation. Freeze–thaw cycles were repeated twice. Subsequently, samples were processed using the APAC protocol (https://d24ci5y4j5ezt1.cloudfront.net/wp/wp-content/uploads/2020/06/M020141.pdf). In brief, we applied Adaptive Focused Acoustics (AFA, Covaris) sonication in the 96-well plate for a total duration of 300 s per column with an LE220-plus Focused ultrasonicator (Covaris) using the following parameters: peak power, 450 W; duty factor, 50%; cycles, 200; average power, 225 W.

In preparation for protein aggregation capture (PAC), magnetic carboxylate-modified particles (Sera-Mag, cat. no. 24152105050350, GE Healthcare/Merck) were washed three times with 1 ml of MS-grade water. Because the protein concentration of the samples varied over a large range, 500 µg of beads were added to each sample well to ensure sufficient beads regardless of the protein concentration. Protein precipitation was induced by the addition of acetonitrile at a final concentration of 70%.

Proteins were subsequently extracted from the solution through precipitation of the magnetic particles and purification by three steps of washing in 2-isopropanol. Following each wash, the plate was placed at 50 °C and shaken at 1,300 rpm for 10 min. To ensure complete precipitation, we incubated the suspension for a further 10 min at room temperature while shaking at 1,300 rpm and then allowed the beads to settle for 10 min without agitation.

To determine the concentration of enzymes needed during sample digestion, we measured the protein yield using a Nanodrop. Samples were then resuspended in digestion buffer, which contained 100 µl of 100 mM Tris (pH 8.5), supplemented with 0.5 µg trypsin and 0.5 µg LysC to achieve an enzyme:protein ratio of 1:20, and incubated overnight at 37 °C with shaking at 1,300 rpm.

Following digestion, the supernatant was removed by placing the 96-well plate on a magnetic rack (DynaMag-96 Side Skirted Magnet, cat. no. 12027, Invitrogen, ThermoFisher Scientific), allowing the supernatant to be easily transferred to a 96-well PCR plate (twin.tec PCR Plate LoBind, semi-skirted, 250 µl; cat. no. 0030129504, Eppendorf). The enzymatic reaction in the collected supernatant was quenched using trifluoracetic acid (TFA) at a final concentration of 1% (v/v). Peptides were purified using two-layer SDB–RPS (Empore SPE Disks; CDS Analytical, cat. no. 98-0604-0226-4) StageTips by three washing steps, twice in 1% TFA in 2-isopropanol and once in 0.2% TFA in water. Following the washing steps, peptides were eluted from the StageTips using elution buffer (80% acetonitrile and 1% NH_4_^+^)^70^. Purified samples were vacuum-dried in a SpeedVac (Eppendorf) at 60 °C for 1.5 h and resuspended in A* injection buffer (2% (v/v) acetonitrile and 0.1% (v/v) TFA in water). Protein concentration was measured in injection buffer for each sample using a Nanodrop, and samples were stored at −20 °C until MS measurement.

### Proteomics UHPLC and mass spectrometry

Samples were analysed using LC–MS instrumentation, comprising an EASY-nLC 1200 ultra-high-pressure system coupled to an Exploris 480 with a nano-electrospray ion source (ThermoFisher Scientific). For each sample, the equivalent of 360 ng of purified peptides was separated on a custom 50-cm C18 LC column^71^. Peptides were eluted from the column using a linear gradient from 10% to 30% buffer B over 90 min at a constant flow rate of 300 nl min^−1^, followed by a stepwise increase of buffer B to 60% for 5 min and an increase to 95% buffer B over the following 5 min. Afterwards, we applied a 5-min wash with 95% buffer B, followed by a decrease to 1% buffer B over 5 min and a 20-min wash.

The column temperature was kept constant at 50 °C using a custom oven, and HPLC parameters were monitored in real time using SprayQC software^72^. MS data were acquired with a Top15 data-dependent MS/MS method. The target values for the full-scan MS spectra were 3 × 10^6^ charges in the *m*/*z* range 300–1,650, with a maximum injection time of 20 ms and a resolution of 60,000 at *m*/*z* 200. Fragmentation of precursor ions was performed by higher-energy C-trap dissociation (HCD) with a normalized collision energy of 27 eV. MS/MS scans were performed at a resolution of 15,000 at *m*/*z* 200 with a target value of 1 × 10^5^ and a maximum injection time of 28 ms. Dynamic exclusion was set to 40 s to avoid repeated sequencing of identical peptides.

A HeLa sample was run approximately every 70 samples to ensure that the performance of the LC system and MS was maintained throughout the entire study. Technical replicates were collected for each plate in a random fashion to assess technical reproducibility. In all, 212 device samples and 56 stool samples passed quality control and were used for analyses (Extended Data Fig. 2a).

### Proteomics data processing

MS raw files were analysed with MaxQuant software (v.2.1.0.0)^73^, and peptide lists were searched against the UniProt human SwissProt and TREMBL FASTA database (version June 2022). A common contaminants database was also included^74^. Our search parameters included cysteine carbamidomethylation as a fixed modification and N-terminal acetylation and methionine oxidation as variable modifications. The false discovery rate (FDR) for proteins and peptides was set to 0 at a minimum peptide length of 7 amino acids. An in silico tryptic digest was used with a maximum of two missed cleavage sites. Peptide identification was performed at a precursor mass accuracy of 7 ppm and a fragment mass accuracy of 20 ppm. A reversed decoy database was used to estimate the fraction of false positive hits. Label-free quantification (LFQ) was performed at a minimum ratio count of 2 (ref. ^75^). LFQ values, or non-normalized intensity values when indicated, were further processed in R (v.4.1.2). Proteins were filtered for 70% valid values in all samples. For PCA, missing values were imputed with the regularized method of the package missMDA (v.1.19), and PCA plots were generated with PCAtools (v.2.4.0). Statistical analysis was performed with limma (v.3.48.3) and a moderated *t*-test with FDR adjustment for multiple-hypothesis testing.

### Sample preparation for LC–MS/MS metabolomics analysis

Supernatants from intestinal samples were extracted using a modified 96-well-plate biphasic extraction^76^. Samples in microcentrifuge tubes were thawed on ice, and 10 µl was transferred to the wells of a 2-ml polypropylene 96-well plate in a predetermined randomized order. A quality-control sample consisting of a pool of many intestinal samples from pilot studies was used to assess analytical variation. A quality-control sample matrix (10 µl) and blanks (10 µl of LC–MS-grade water) were included for every tenth sample. Further, 170 µl of methanol containing UltimateSPLASH Avanti Polar Lipids was added to each well as an internal standard. Then, 490 µl of methyl-*tert*-butyl-ether (MTBE) containing the internal standard cholesterol ester 22:1 was added to each well. Plates were sealed, vortexed vigorously for 30 s and shaken on an orbital shaking plate for 5 min at 4 °C. The plate was unsealed, and 150 µl of cold water was added to each well. Plates were resealed, vortexed vigorously for 30 s and centrifuged at 4,000 RCF for 12 min at 4 °C.

From the top phase of the extraction wells, two aliquots of 180 µl each were transferred to new 96-well plates, and two aliquots of 70 µl each from the bottom phase were transferred to two other new 96-well plates. Plates were spun in a rotary vacuum until dry, sealed and stored at −80 °C until LC–MS/MS analysis. One of the 96-well plates containing the aqueous phase of extract was dissolved in 35 µl of HILIC-run solvent (8:2 acetonitrile/water). Five microlitres was analysed using non-targeted HILIC LC–MS/MS analysis. The autosampler temperature was kept at 4 °C. Immediately after HILIC analysis, the 96-well plates were spun in a rotary vacuum until dry, sealed and stored at −80 °C until targeted bile acid analysis.

Multiple dilutions were prepared for bile acid analysis as follows. The dried samples described above were dissolved in 60 µl of bile acid-run solvent (1:1 acetonitrile/methanol containing six isotopically labelled bile acid standards at 100 ng ml^–1^) by 30 s of vortexing and 5 min of shaking on an orbital shaker. From this plate, 5 µl was transferred to a new 96-well plate and combined with 145 µl of bile acid-run solvent. Both dilutions were analysed for all samples, and samples that still presented bile acids above the highest concentration of the standard curve (1,500 ng ml^–1^) were diluted 5:145 once more and re-analysed. A nine-point standard curve that ranged from 0.2 ng ml^–1^ to 1,500 ng ml^–1^ was used with all samples. The standard curve solutions were created by drying bile acid standard solutions to achieve the desired mass of bile acid standards and then dissolved in bile acid-run solvent. Three standard curve concentration measurements were analysed after every 20 samples during data acquisition along with one method blank.

For stool analyses, approximately 4 mg (±1 mg) of wet stool was transferred to 2-ml microcentrifuge tubes. Twenty microlitres of quality-control mix was added to the microcentrifuge tubes for quality-control samples. Blank samples were generated using 20 µl of LC–MS-grade water. To each tube, 225 µl of ice-cold methanol containing internal standards (as above) was added, followed by 750 µl of ice-cold MTBE with cholesterol ester 22:1. Two 3-mm stainless steel grinding beads were added to each tube, and tubes were processed in a Geno/Grinder automated tissue homogenizer and cell lyser at 1,500 rpm for 1 min. Then, 188 µl of cold water was added to each tube. Tubes were vortexed vigorously and centrifuged at 14,000 RCF for 2 min. Two aliquots of 180 µl each of the MTBE layer and two aliquots of 50 µl each of the lower layer were transferred to four 96-well plates, and the plates were spun in a rotary vacuum until dry, sealed and stored at −80 °C until analysis with the intestinal samples. Stool samples were analysed using HILIC non-targeted LC–MS/MS and diluted in an identical manner to intestinal samples as described above. Stool samples were analysed in a randomized order after intestinal samples.

### Metabolomics data acquisition

Samples were analysed using a Vanquish UHPLC system coupled to a TSQ Altis triple-quadrupole mass spectrometer (ThermoFisher Scientific). An Acquity BEH C18 column (1.7 µm, 2.1 mm × 100 mm) with an Acquity BEH C18 guard column (1.7 µm, 2.1 mm × 5 mm) was used for chromatographic separation with mobile phases A (LC–MS-grade water with 0.1% formic acid) and B (LC–MS-grade acetonitrile with 0.1% formic acid) and with a flow rate of 400 µl min^–1^ and column temperature of 50 °C. The gradient began at 20% B for 1 min and shifted to 45% B between 1 and 11 min, to 95% B between 11 and 14 min and to 99% B between 14 and 14.5 min; 99% B was maintained until 15.5 min and transitioned to 20% B between 15.5 and 16.5 min; and 20% B was maintained until 18 min. The autosampler temperature was kept at 4 °C. The injection volume was 5 µl, and MRM scans were collected for all bile acids and internal standards (Supplementary Table 6).

### Metabolomics data processing

MRM scans were imported to Skyline^77^ software. Skyline performed peak integration for all analytes with given mass transitions and retention time windows optimized using authentic chemical standards (Supplementary Table 6). The chromatogram for each analyte was manually checked to confirm correct peak integration. Peak area was exported for all analytes. Analytes were omitted from further analysis if a convincing chromatographic peak was not observed in ≥1 sample (Supplementary Table 6). The ratio of analyte to its closest eluting internal standard was calculated and used for quantification. A linear model was fit to standard curve points for each bile acid (*R*^2^ > 0.995 for all bile acids), and the model was applied to all samples and blanks to calculate concentrations. The average concentration reported for method blanks was subtracted from sample concentrations. Because multiple dilutions were analysed for each sample, the measurement closest to the centre of the standard curve (750 ng ml^–1^) was used. Zero values were imputed with a concentration value between 0.001 and 0.1 ng ml^–1^. Concentrations were reported as ng ml^–1^ for intestinal sample liquid supernatant and ng g^–1^ for wet stool. In all, 218 device samples and 57 stool samples passed quality control and were used for analyses (Extended Data Fig. 2a).

### Non-targeted bile acid quantification

**Extended Data Fig. 9:**
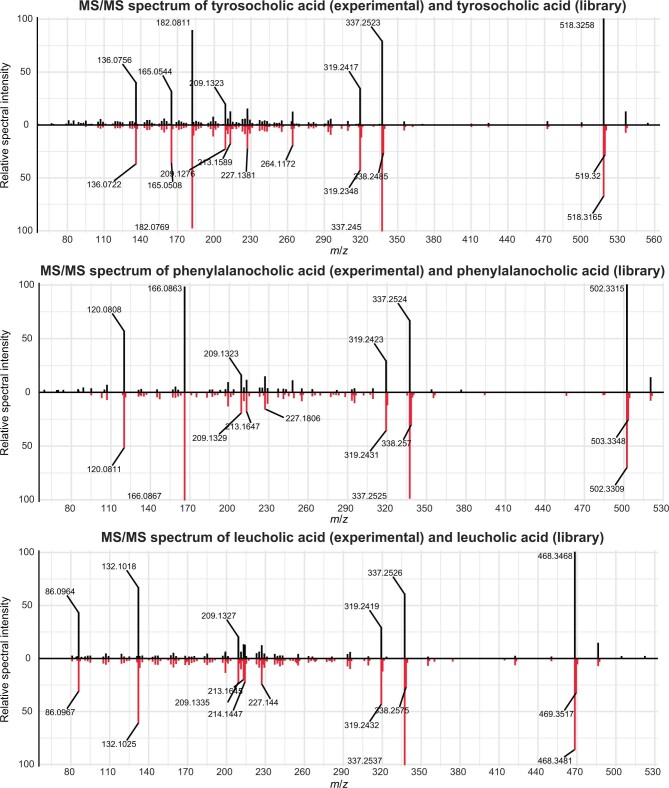
Microbially conjugated bile acid identification. Head-to-tail matches of experimental (top) to library (bottom) spectra from bile acids conjugated to tyrosine, phenylalanine, and leucine.

Bile acids conjugated to amino acids (for example, TyroCA, LeuCA and PhenylCA) were not included in the list for targeted analysis. Nonetheless, 22 microbially conjugated bile acids were detected during non-targeted data acquisition for intestinal and stool samples using HILIC chromatography as described previously^78^. Peaks corresponding to these microbially conjugated bile acids were annotated using *m*/*z* values for precursor mass, diagnostic MS/MS fragment ions (337.2526 for trihydroxylated and 339.2682 for dihydroxylated bile acids) and the corresponding amide conjugate fragment ion (Supplementary Table 7), as reported previously^40^. MS/MS spectra from synthetic standards for three microbially conjugated bile acids (Extended Data Fig. 9) served as positive controls based on previously collected experimental MS/MS spectra^35^. Non-targeted HILIC analysis did not include bile acid standard curves to allow for direct quantification, so approximate quantification was achieved by comparing the concentration of GCA from targeted analysis to GCA peak height intensity from non-targeted analysis. A quadratic model was fit to GCA values from both analyses (*R*^2^ = 0.89) and applied to the peak height intensity values of microbe-conjugated bile acids to calculate their approximate concentration. Approximate concentrations were used for analysis of bile acids measured with non-targeted analysis.

### Reporting summary

Further information on research design is available in the Nature Portfolio Reporting Summary linked to this article.

## Online content

Any methods, additional references, Nature Portfolio reporting summaries, source data, extended data, supplementary information, acknowledgements, peer review information; details of author contributions and competing interests; and statements of data and code availability are available at 10.1038/s41586-023-05989-7.

## Supplementary information

Reporting Summary

Supplementary Tables This file contains Supplementary Tables 1–7.

Supplementary Video 1 Devices targeting the jejunum and ascending colon were connected to a capsule endoscope, enabling visualization of successful in vivo sampling in a human participant.

Supplementary Video 2 Time-lapse imaging of a sample retrieved from a device. The field of view was biasedly chosen to contain one human epithelial cell.

Supplementary Video 3 Time-lapse imaging of a fresh stool sample.

## Data Availability

The 16S rRNA and metagenomics sequencing reads are available on NCBI under BioProject PRJNA822660. The mass spectrometry proteomics datasets are available through the ProteomeXchange Consortium in the PRIDE^79^ partner repository with dataset identifier PXD038906. The targeted and non-targeted bile acid metabolomics datasets are available on Metabolomics Workbench under project numbers ST002073 and ST002075. The minimum datasets necessary for reproduction of figures or extended research related to this article are available on GitHub at https://github.com/jgrembi/capscan-profiling-human-intestine.

## Code availability

Custom code for the generation of figures and statistical calculations in this manuscript is available on Zenodo at 10.5281/zenodo.7683655 (ref. ^80^) and GitHub at https://github.com/jgrembi/capscan-profiling-human-intestine.
