# Supplementary material for: Profiling the human intestinal environment under physiological conditions
Source: Nature. 2023 May 10;617(7961):581–91. doi: 10.1038/s41586-023-05989-7 (PMC10191855; doi:10.1038/s41586-023-05989-7)
Supplement: Supplementary file 1 — Reporting Summary [file 41586_2023_5989_MOESM1_ESM.pdf]

nature portfolio

Corresponding author(s): *Double-blind peer review submissions: write DBPR and your manuscript number here instead of author names.*

Last updated by author(s): *YYYY-MM-DD*

Reporting Summary

Nature Portfolio wishes to improve the reproducibility of the work that we publish. This form provides structure for consistency and transparency in reporting. For further information on Nature Portfolio policies, see our Editorial Policies and the Editorial Policy Checklist.

Statistics

For all statistical analyses, confirm that the following items are present in the figure legend, table legend, main text, or Methods section.

n/a	Confirmed	
☐	☒	The exact sample size (*n*) for each experimental group/condition, given as a discrete number and unit of measurement
☐	☒	A statement on whether measurements were taken from distinct samples or whether the same sample was measured repeatedly
☐	☒	The statistical test(s) used AND whether they are one- or two-sided<br>*Only common tests should be described solely by name; describe more complex techniques in the Methods section.*
☐	☒	A description of all covariates tested
☐	☒	A description of any assumptions or corrections, such as tests of normality and adjustment for multiple comparisons
☐	☒	A full description of the statistical parameters including central tendency (e.g. means) or other basic estimates (e.g. regression coefficient) AND variation (e.g. standard deviation) or associated estimates of uncertainty (e.g. confidence intervals)
☐	☒	For null hypothesis testing, the test statistic (e.g. *F*, *t*, *r*) with confidence intervals, effect sizes, degrees of freedom and *P* value noted<br>*Give P values as exact values whenever suitable.*
☐	☒	For Bayesian analysis, information on the choice of priors and Markov chain Monte Carlo settings
☐	☒	For hierarchical and complex designs, identification of the appropriate level for tests and full reporting of outcomes
☐	☒	Estimates of effect sizes (e.g. Cohen's *d*, Pearson's *r*), indicating how they were calculated

Our web collection on statistics for biologists contains articles on many of the points above.

Software and code

Policy information about availability of computer code

Data collection	The following software was used to collect and analyze data: µManager v. 1.4, R v. 4.0.3, DADA2 v. 4.0.2, phyloseq v. 4.0.0, topicmodels v. 4.0.0, sangeranalysesR v. 4.0.0, Skewer v. 0.2.2, Bowtie2 v. 2.4.1, MEGAHIT v. 1.2.9, MetaBAT 2 v. 2.15, checkM v. 1.1.3, quast v. 5.0.2, dRep v. 3.0.0, Prodigal v. 2.6.3, run_dbcan.py v. 3.0.5, rgi v. 5.2.0, VirSorter2, DeepVirFinder, VIBRANT, PRopagAtE, MaxQuant v. 2.1.0.0, missMDA v. 1.19, PCAtools v. 2.4.0, limma v. 3.48.3, and Skyline.
Data analysis	Custom R (v. 4.0.3) scripts were used for sequencing analysis, statistical tests, and generation of all figures. A Github repository with all scripts is available (https://github.com/jgrembi/capscan-profiling-human-intestine), and it's availability is stated in the manuscript.

For manuscripts utilizing custom algorithms or software that are central to the research but not yet described in published literature, software must be made available to editors and reviewers. We strongly encourage code deposition in a community repository (e.g. GitHub). See the Nature Portfolio guidelines for submitting code & software for further information.

Data

Policy information about availability of data

All manuscripts must include a data availability statement. This statement should provide the following information, where applicable:

- Accession codes, unique identifiers, or web links for publicly available datasets
- A description of any restrictions on data availability
- For clinical datasets or third party data, please ensure that the statement adheres to our policy

The 16S and metagenomics sequencing reads are available on NCBI under BioProject PRJNA822660. The mass spectrometry proteomics datasets are available on ProteomeXchange Consortium via the PRIDE partner repository with the dataset identifier PXD038906. The targeted and untargeted bile acid metabolomics datasets are available on Metabolomics Workbench under project numbers ST002073 and ST002075.

Human research participants

Policy information about studies involving human research participants and Sex and Gender in Research.

Reporting on sex and gender	We have reported subjects' sex (n=15, 8 female and 7 male) in the supplemental table 1 of the manuscript. Our findings did not investigate sex-based differences. We did not document subjects' gender in this study.
Population characteristics	Age ranges were from 22-64, mean = 42. No underlying medical conditions were reported.
Recruitment	Subjects were recruited based on those who knew the researchers or were affiliated with the researchers in this study in some manner, thus recruitment was biased toward individuals living in and around Palo Alto, CA. Many participants in the study are scientists employed at Stanford University. No public notice was posted about the study.
Ethics oversight	The study was approved by the WIRB-Copernicus Group IRB (study #1186513).

Note that full information on the approval of the study protocol must also be provided in the manuscript.

Field-specific reporting

Please select the one below that is the best fit for your research. If you are not sure, read the appropriate sections before making your selection.

☒ Life sciences ☐ Behavioural & social sciences ☐ Ecological, evolutionary & environmental sciences

For a reference copy of the document with all sections, see nature.com/documents/nr-reporting-summary-flat.pdf

Life sciences study design

All studies must disclose on these points even when the disclosure is negative.

Sample size	15 individuals were selected. As this study was the first human study for this device, we aimed to have a large enough sample size to draw general statistical conclusions, with the precaution that the device had not been widely utilized or tested. Extended Data Fig. 2 details the collection of 344 human microbiome samples, and a total of 297 samples were analyzed after filtering for successful collection and sequencing depth.
Data exclusions	Individuals with any major medical conditions, especially those that are relevant to transit along the human gastrointestinal tract, were excluded from this study.
Replication	Subject 1 swallowed 4 of each device type twice (n=16) as part of an effort to test reproducibility. Fig. 2 shows how technical variability was lower than temporal, spatial, and temporal+spatial variability.
Randomization	There was no experimental group in this study.
Blinding	Data collection and analysis was blind to the researchers (i.e. the researchers were unaware of any identifying information of the research participants, including age and sex).

Reporting for specific materials, systems and methods

We require information from authors about some types of materials, experimental systems and methods used in many studies. Here, indicate whether each material, system or method listed is relevant to your study. If you are not sure if a list item applies to your research, read the appropriate section before selecting a response.

Materials & experimental systems

n/a	Involved in the study
☒	☐ Antibodies
☒	☐ Eukaryotic cell lines
☒	☐ Palaeontology and archaeology
☒	☐ Animals and other organisms
☒	☐ Clinical data
☒	☐ Dual use research of concern

Methods

n/a	Involved in the study
☒	☐ ChIP-seq
☒	☐ Flow cytometry
☒	☐ MRI-based neuroimaging
